# Circular RNA FTO functions as a hsa-miR-141-3p sponge to regulate the growth and migration abilities of human retinal endothelial cells via up-regulating *ZEB1*

**DOI:** 10.1371/journal.pone.0338208

**Published:** 2025-12-05

**Authors:** Yaoyao Chen, Renjian Hu, Enhui Li, Gaochun Li, Bing Xia, Jie Zhou

**Affiliations:** 1 Department of Ophomology, Tiantai People’s Hospital of Zhejiang Province, Tiantai County, Zhejiang Province, China; 2 Department of Ophomology, Affiliated Hangzhou First People’s Hospital, Zhejiang University School of Medicine, Hangzhou City, Zhejiang Province, China; 3 Department of Ophomology, Taizhou Hospital of Zhejiang Province affiliated to Wenzhou Medical University, Lin Hai County, Zhejiang Province, China; Longgang Otorhinolaryngology Hospital & Shenzhen Key Laboratory of Otorhinolaryngology, Shenzhen Institute of Otorhinolaryngology, CHINA

## Abstract

Proliferative diabetic retinopathy (PDR) is a microvascular complication of diabetes mellitus. Circular RNAs have been implicated in the pathogenesis of PDR. This study aimed to elucidate the specific mechanism by which circFTO contributes to PDR progression. circFTO expression was significantly upregulated in PDR patients and in high glucose (HG)-treated human retinal endothelial cells (HRECs). Knockdown of circFTO suppressed cell proliferation, migration, and tube formation in HG-treated HRECs. Furthermore, hsa-miR-141-3p levels were downregulated, while ZEB1 levels were upregulated in HG-treated HRECs. Dual-luciferase reporter assays demonstrated that hsa-miR-141-3p directly interacts with both circFTO and ZEB1. Additionally, hsa-miR-141-3p silencing reversed the effects of circFTO knockdown, and ZEB1 overexpression counteracted the effects of hsa-miR-141-3p mimic transfection. These findings suggest that circFTO promotes PDR progression via the hsa-miR-141-3p/ZEB1 axis. Collectively, our findings provide preliminary mechanistic insights into the role of circFTO in PDR progression, suggesting its potential as a candidate for further investigation as a diagnostic biomarker or therapeutic target.

## Introduction

Diabetic retinopathy (DR) is one of the major ophthalmic diseases threatening populations worldwide [1,2]. The incidence rate of diabetes is increasing annually, and the progression of DR has also been exacerbated [3]. DR consists of nonproliferative and proliferative subtypes [4]. Proliferative DR (PDR) represents the advanced stage of DR, characterized by retinal ischemia, hypoxia, abnormal neovascularization, vitreous hemorrhage, and tractional retinal detachment. PDR is the primary cause of nontraumatic vitreous hemorrhage, which can seriously endanger patients’ vision and even lead to blindness [5]. Previous studies have confirmed that excessive proliferation is an important cause of PDR [6,7]. Hyperglycemia induces the excessive release of inflammatory factors in retinal tissue, ultimately leading to retinal injury [8]. In addition, the pathogenesis of DR involves a complex process regulated by multiple genes and molecular factors [9]. Numerous protein-coding genes associated with hyperglycemia have been investigated during PDR progression [10–12]. However, the underlying mechanisms of PDR still require further investigation.

Circular RNAs (circRNAs) belong to a class of non-coding RNA molecules. The 3′ end and 5′ end are covalently linked through alternative splicing of precursor mRNA to form a backsplicing closed-loop structure [13,14]. CircRNAs were first observed in RNA viruses using electron microscopy in 1976 and were subsequently identified in diverse organisms, including animals and other species [15,16]. Recent studies have demonstrated that circRNAs participate in numerous pathophysiological processes, including autophagy, apoptosis, and proliferation in vivo, and are involved in regulating the pathological mechanisms of neurological, cardiovascular, and tumor diseases [17–19]. Moreover, it has been established that circRNAs are closely associated with various diabetes-related complications, such as diabetic nephropathy [20], diabetes-associated cardiovascular disease [21], diabetic peripheral neuropathy [22] and DR [23]. Investigating the role of circRNAs in DR microvascular lesions may provide novel biomarkers for the management of these conditions. He et al. [24] reported that circFTO levels were significantly elevated in patients with PDR. However, the regulatory mechanisms by which circFTO contributes to PDR progression remain unclear.

MicroRNA-141-3p (hsa-miR-141-3p) is a key regulator of post-transcriptional gene expression, functioning by binding to complementary sequences in the 3′ untranslated regions (UTRs) of target mRNAs, thereby suppressing translation or promoting mRNA degradation [25] Emerging evidence has highlighted its role in modulating vascular biology and inflammatory responses, particularly in pathological conditions involving endothelial dysfunction. In vascular contexts, hsa-miR-141-3p has been shown to inhibit pathological angiogenesis and inflammation by targeting genes such as NLRP3 (a central component of the inflammasome) [26] and HMGB1 (a pro-inflammatory mediator) [27]. Similarly, in osteoarthritis [28] and endometriosis [29], hsa-miR-141-3p suppresses fibrosis and ectopic tissue proliferation by targeting genes that regulate cell survival and migration. In glaucoma, hsa-miR-141-3p inhibits the proliferation of retinal endothelial cells, suppresses angiogenesis, and promotes apoptosis of retinal ganglion cells [30]. However, the role of hsa-miR-141-3p in PDR remains unclear.

Zinc Finger E-box Binding Homeobox 1 (ZEB1) is a transcription factor that plays a pivotal role in epithelial-mesenchymal transition (EMT), tumor progression, and vascular remodeling [31]. ZEB1 exerts its regulatory effects by repressing epithelial genes (e.g., E-cadherin) and activating mesenchymal and pro-migratory pathways, while also modulating angiogenesis through the upregulation of vascular endothelial growth factor A (VEGFA) [32]. In endothelial cells, ZEB1 enhances sprouting angiogenesis and increases vascular permeability, processes critical to both physiological repair mechanisms and pathological conditions such as cancer and fibrosis [33]. Notably, ZEB1’s involvement in vascular biology extends to diabetic complications: in retinal endothelial cells exposed to hyperglycemic conditions, ZEB1 upregulation may exacerbate PDR by promoting aberrant vessel formation via VEGFA-driven angiogenesis and EMT-like phenotypes [34]. This observation aligns with findings demonstrating that elevated ZEB1 expression in endothelial cells correlates with impaired barrier integrity and enhanced migratory activity, hallmarks of diabetic retinopathy progression. Collectively, ZEB1’s dual capacity to regulate cellular plasticity and angiogenic processes positions it as a central mediator of both physiological homeostasis and pathogenic mechanisms in endothelial-dependent disorders.

Therefore, in this study, we predicted and validated that circFTO functions as an hsa-miR-141-3p sponge RNA to regulate ZEB1 expression in PDR using bioinformatics tools. To investigate the functional role of circFTO in PDR, we established an in vitro cellular model of PDR.

## Materials and methods

### Patients and specimens

Sixty patients were recruited from Taizhou Hospital of Zhejiang Province and divided into two groups: PDR group: Retinal fibrovascular membranes were collected from 30 patients with PDR through pars plana vitrectomy. Non-PDR group: Retinal fibrovascular membranes were collected from 30 Non-PDR patients through pars plana vitrectomy. In addition, aqueous humor samples from 30 non-diabetic controls (cataract patients) were collected as the Control group via cataract surgery. This study was performed according to the principles of the Declaration of Helsinki (DOH, 2013 version) and the Association for Research in Vision and Ophthalmology (ARVO) statement for research involving human subjects. The research was agreed by the Ethical Review Committee of the Taizhou Hospital of Zhejiang Province (No. K20211233). Informed consent was obtained from all individual participants included in the study. This study was started in January 1, 2022 and ended in – December 1, 2022.

### Cell culture

Human retinal microvascular endothelial cells (HRECs) were obtained from Innoprot® (P10880; Derio–Bizkaia, Spain). To induce cell injury, 25 mmol/L glucose (HG) was added to the cells for 48 h, whereas 5.5 mmol/L glucose was used as the normal glucose (NG) control group [35]. To equalize the osmotic pressure between the 5.5 mmol/L and 25 mmol/L glucose media, 19.5 mmol/L mannitol was added to the 5.5 mmol/L glucose medium. All cells were cultured in ECM medium (300 mM osmotic pressure) supplemented with 10% FBS and 1% penicillin-streptomycin for 4 weeks.

### Cell transfection

siRNA targeting circFTO (si-circFTO 1#, si-circFTO 2#) or si-NC, hsa-miR-141-3p inhibitor or inhibitor NC, hsa-miR-141-3p mimic or mimic NC, ZEB1 overexpression plasmid (ZEB1), and vector control were obtained from GenePharma (Shanghai, China). Subsequently, all these plasmids were transfected into HRECs using Lipofectamine® 2000 transfection reagent. After transfection, HRECs were cultured in preparation for subsequent experiments. The sequences of si-circFTO 1#, si-circFTO 2#, hsa-miR-141-3p inhibitor or si-nc and inhibitor nc were shown in Table 1.

**Table 1:** Sequences of siRNAs and inhibitor.

	Sequences (5’-3’)
Si-circFTO 1#	ATGGAGGGTGTGATGATCTCA
Si-circFTO 2#	GGAGGGTGTGATGATCTCAAT
Si-nc	UACUGUGCAUCAUCUGGUUUC
hsa-miR-141-3p inhibitor	CCAUCUUUACCAGACAGUGUUA
inhibitor nc	UCUCCUCUCAACAGGGUAAUAU

### Cell viability assay

Cell viability was measured using an MTT assay after treating each group of cells for 72 hours. The procedure was as follows: the cells were cultured in 96-well culture plate (2 × 10^3^ cells per well). Then 20 μl MTT (5 g/L, Invitrogen company) were added to the cells and maintained for 4 h. Next, 150 μl DMSO (Invitrogen) was supplemented. The absorbance (A) value was detected at 490 nm with a microtiter reader (Tecan Infinite 200, Switzerland).

### Cell migration assay

HRECs were seeded into the upper chamber of the Transwell system coated with Matrigel. The lower chamber was filled with culture medium supplemented with 10% FBS. After 24 hours, 4% paraformaldehyde was added to fix the migrated HRECs, followed by staining with 0.1% crystal violet. Finally, the migrated cells were visualized and counted using a light microscope in five randomly selected fields.

### Tube formation assay

The serum-free medium was mixed with Matrigel matrix in a 1:1 ratio. The mixture (50 µL/well) was added to a 96-well plate and incubated at 37 °C for 60 minutes. HRECs were trypsinized with 0.25% trypsin, counted, resuspended, and adjusted to a density of 3 × 10⁵ cells/mL. The cells were then seeded into 96-well plates pre-coated with Matrigel and incubated for 20 hours. Tubular structures formed by the HRECs were analyzed using a light microscope in five randomly selected fields.

### Real-Time quantitative polymerase chain reaction (RT-qPCR)

All RNA from HRECs and tissues was extracted using TRIzol® (Invitrogen). cDNA was synthesized using the PrimeScript™ RT Reagent Kit (Takara Bio Inc.). For miR-141-3p-specific reverse transcription, a stem-loop primer-based strategy was employed to enhance specificity and sensitivity. Briefly, 1 µg of total RNA was reverse-transcribed into cDNA using the Mir-X™ miRNA First-Strand Synthesis Kit (Takara Bio Inc.). This method utilizes a stem-loop primer complementary to the 3’ end of miR-141-3p, enabling selective priming and efficient cDNA synthesis of mature miRNAs. Subsequently, RT-qPCR was performed using SYBR Premix Ex Taq™ (Takara Bio Inc.) on a CFX96 Real-Time PCR Detection System (Bio-Rad). The reaction conditions included a 20 µL reaction volume with the following thermal profile: 95 °C for 30 seconds, followed by 40 cycles of 95 °C for 5 seconds and 60 °C for 30 seconds. U6 and GAPDH were used as internal controls. RT-qPCR results were quantified using the 2^-ΔΔCt^ method as previously described [36]. The sequences of primers were were shown in Table 2.

**Table 2:** Sequences of primers.

Primers (5’-3’)	Forward	Reverse
CircFTO	TCGGTGGGTGGAACTAAA	GTGGAAGAAGATGGAGGGT
hsa-miR-141-3p	GCCGAGTAACACTGTCTGGT	CTCAACTGGTGTCGTGGAGT
ZEB1	GATGATGAATGCGAGTCAGATGC	ACAGCAGTGTCTTGTTGTTGT
U6	CTCGCTTCGGCAGCACA	AACGCTTCACGAATTTGCGT
GAPDH	GACAGTCAGCCGCATCTTCT	GCGCCCAATACGACCAAATC

### CircFTO stability determination

For RNase R treatment, HRECs were harvested at 80% confluency and subjected to RNase R digestion (3 U/μg RNA, Epicentre, USA) for 30 minutes at 37°C in the presence of RNase R buffer. The reaction was terminated by adding 200 μL of TRIzol reagent (Invitrogen, USA), and total RNA was extracted according to the manufacturer’s instructions. Then, RT-qPCR was performed to detect the expression of circFTO or linear FTO.

For Actinomycin D treatment, HRECs were treated 2 mg/ml of Actinomycin D (Sigma-Aldrich, Shanghai, China) with dimethyl sulfoxide (DMSO) treatment as the negative control. The RNA expression levels of circFTO and linear FTO were detected by RT-qPCR at 4, 8, 12 and 24 h.

### Dual-luciferase reporter

The predicted interactions between circFTO/ZEB1 and hsa-miR-141-3p were validated using bioinformatics tools. Wild-type (WT) and mutant-type (MUT) sequences of circFTO or ZEB1 were cloned into the pmiR-RB-Report™ luciferase reporter vector. The hsa-miR-141-3p mimic, along with WT or MUT 3’-UTR reporter constructs of circFTO or ZEB1, were co-transfected into HRECs. After 48 hours, the Dual Luciferase Reporter Assay Kit (Beyotime, Nantong, China) was used to measure relative luciferase activity.

### Western blot

Total protein was extracted from HRECs using RIPA lysis buffer (Beyotime, China) supplemented with protease inhibitor cocktail (Roche, Switzerland). Protein concentration was determined using the BCA Protein Assay Kit (Beyotime, China). Equal amounts of protein (30 µg per lane) were separated by 10% sodium dodecyl sulfate-polyacrylamide gel electrophoresis (SDS-PAGE). The electrophoresis was performed initially at 80 V for 30 minutes while the samples migrated through the stacking gel, followed by 120 V for approximately 60−70 minutes until the dye front reached the bottom of the gel. Subsequently, the separated proteins were transferred onto polyvinylidene difluoride (PVDF) membranes (Millipore, USA) using a wet transfer system at a constant current of 300 mA for 90 minutes. To confirm equal protein loading and transfer efficiency, the membranes were briefly stained with Ponceau S solution (Beyotime, China) prior to immunoblotting. Following destaining, the membranes were blocked with 5% (w/v) skim milk powder (BD Biosciences, USA) in Tris-buffered saline containing 0.1% Tween-20 (TBST) for 1 hour at room temperature. The membranes were then incubated overnight at 4 °C with the following primary antibodies diluted in TBST containing 5% BSA: anti-E-cadherin (1:1000, Cat. No. ab1416, Abcam), anti-N-cadherin (1:1000, Cat. No. ab18203, Abcam), anti-Vimentin (1:1500, Cat. No. ab92547, Abcam), anti-ZEB1 (1:1000, Cat. No. ab180905, Abcam) and anti-GAPDH (1:5000, Cat. No. ab8245, Abcam). GAPDH served as the loading control. The specificity of these antibodies has been previously validated by the manufacturer and in peer-reviewed literature for use in human endothelial cells. After washing with TBST, the membranes were incubated with a horseradish peroxidase (HRP)-conjugated goat anti-rabbit (for E-cadherin, N-cadherin, Vimentin) or goat anti-mouse (for GAPDH) secondary antibody (1:2000, Cat. No. ab6721 and ab6789, respectively; Abcam) at room temperature for 2 hours. Protein bands were visualized using an enhanced chemiluminescence (ECL) detection kit (Millipore, USA) and imaged with a ChemiDoc™ Imaging System (Bio-Rad, USA).

### Statistical analysis

Statistical analysis was conducted using SPSS 21.0 software (IBM Corp.). Each experiment was independently repeated three times. Clinical data and cell-based experimental results followed a normal distribution, as confirmed by the Shapiro-Wilk test. Data from this study were expressed as mean ± standard deviation (SD). Comparisons between two groups were performed using an unpaired Student’s t-test, while differences among multiple groups were analyzed by one-way ANOVA followed by post-hoc Tukey’s test. A p-value of < 0.05 was considered statistically significant.

## Results

### The circFTO level was elevated in PDR

**Fig 1:**
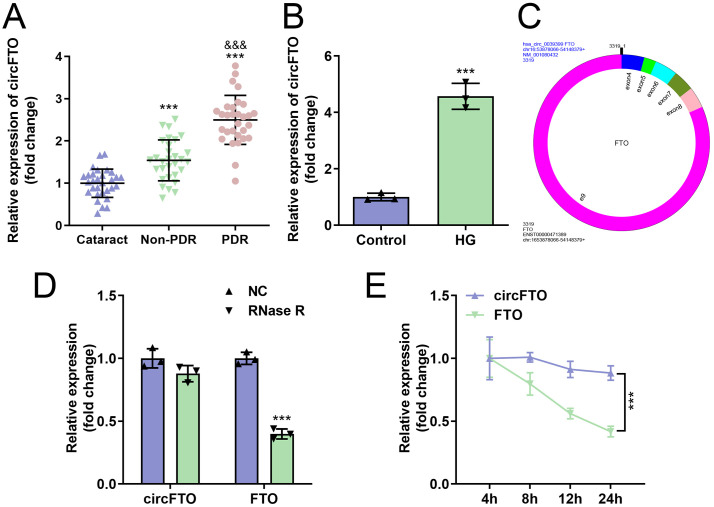
The *circFTO* level was elevated in PDR. The *circFTO* levels in cataract patients, non-PDR and PDR patients (A) and HG treated HRECs (B) were determined with RT-qPCR. ***P<0.001 VS cataract group. &&&P<0.001 VS non-PDR group. (C) The genomic locus and sequence of circFTO. RT-qPCR was performed to detect the expressions of liner FTO and liner FTO after RNase R (D) and Actinomycin D (E) treatment. (A) The differences were detected using One-way ANOVA (n=30). (B, D, E) The differences were detected using Student-T test (n=3).

First, circFTO expression in PDR patients and HG-treated HRECs was quantified using RT-qPCR. We observed significantly elevated circFTO levels in retinal proliferative fibrovascular membranes from PDR patients compared to those in non-PDR patients and cataract control groups (Fig 1A). Furthermore, circFTO expression was also upregulated in HRECs exposed to high-glucose conditions (Fig 1B). The genomic locus and sequence of circFTO are illustrated in Fig 1C. In addition, RNase R treatment significantly decreased liner FTO expressions, while showed no effects on circFTO expressions (Fig 1D). Actinomycin D treatment significantly decreased the RNA stability of liner FTO compared with circFTO (Fig 1E).

### circFTO-silenced reduced the biological behavior of the HRECs

**Fig 2:**
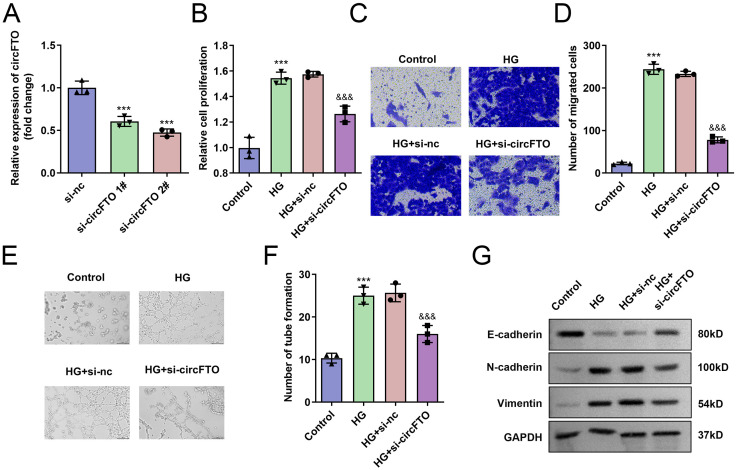
*circFTO*-silenced inhibited the biological behavior of the HRECs. A: Transfection efficiency of si-*circFTO* 1# and si-*circFTO* 2#. B: Cell proliferation was dected with MTT assay. C: The cell migration was determined with Transwell assay. D: Number of tube formation was determined. E: The protein expressions of E-cadherin, N-cadherin and Vimentin were determined with western blot. **P<0.01, ***P<0.001 VS Control group. &&P<0.01 VS HG+si-nc group. The differences were detected using One-way ANOVA (n=3).

Next, HRECs were transfected with si-circFTO 1# and si-circFTO 2#. CircFTO expression was significantly reduced post-transfection (Fig 2A), with si-circFTO 2# demonstrating greater knockdown efficiency. Consequently, si-circFTO 2# was selected for subsequent experiments. Proliferation, migration, and tube formation were markedly increased in the HG group compared to controls. However, circFTO silencing significantly suppressed these processes in HG-treated HRECs (Fig 2B–2D). Furthermore, in the HG group, E-cadherin protein levels were decreased, while N-cadherin and Vimentin were upregulated. In contrast, circFTO knockdown reversed these effects by increasing E-cadherin expression and decreasing N-cadherin and Vimentin levels in HG-treated HRECs (Fig 2E).

### circFTO sponged to hsa-miR-141-3p in HRECs

**Fig 3:**
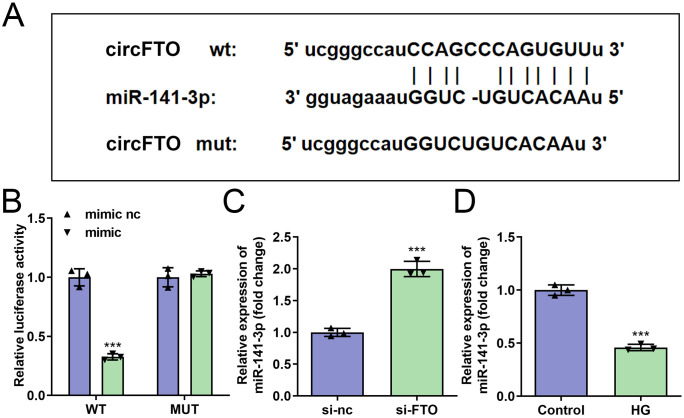
*circFTO* serves as a miRNA sponge for *hsa-miR-141-3p* in HRECs. A-B: The target *hsa-miR-141-3p* and binding sites of *circFTO* was predicted by starbase software and validated by the dual-luciferase reporter. The *hsa-miR-141-3p* expression in HRECs was determined by RT-qPCR after si-*circFTO* (C) and HG (D) treatment. The differences were detected using Student-T test (n=3).

Using the starBase database (https://rnasysu.com/encori/), we identified multiple miRNAs potentially targeting circFTO. Among these, hsa-miR-141-3p has been shown to be closely associated with the growth and development of vascular endothelial cells [30]. Consequently, hsa-miR-141-3p was selected for further investigation. The predicted binding sites between circFTO and hsa-miR-141-3p are illustrated in Fig 3A. Co-transfection of the circFTO 3’UTR-WT construct with the hsa-miR-141-3p mimic significantly reduced luciferase activity compared to the mimic NC group (Fig 3B). Furthermore, circFTO knockdown markedly upregulated hsa-miR-141-3p levels in HRECs (Fig 3C). Notably, hsa-miR-141-3p expression was decreased in HG-treated HRECs (Fig 3D).

### hsa-miR-141-3p-silenced abrogated si-circFTO effects in HRECs

**Fig 4:**
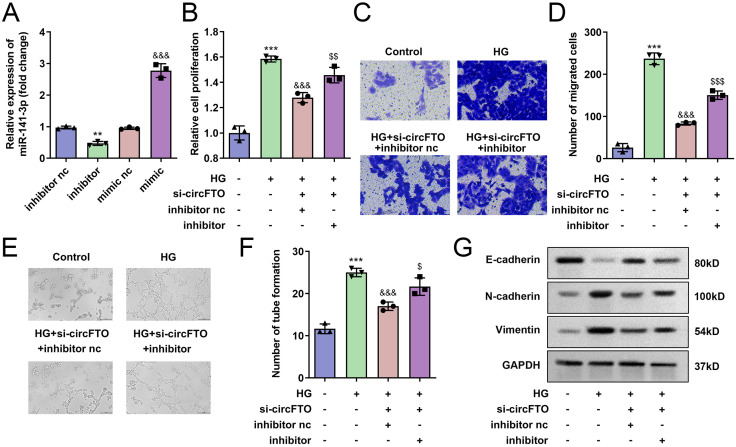
*hsa-miR-141-3p*-silenced abrogated the si-circFTO effects in HRECs. A: Transfection efficiency of *hsa-miR-141-3p* inhibitor and *hsa-miR-141-3p* mimic. B: Cell proliferation was detected using MTT assay. C: The cell migration was determined with Transwell assay. D: Number of tube formation was determined. E: The protein expressions of E-cadherin, N-cadherin and Vimentin were determined with western blot. **P<0.01, ***P<0.001 VS Control group. &P<0.05, &&P<0.01, &&&P<0.001 VS HG group. $P<0.05 VS HG+si-*circFTO*+inhibitor nc group. The differences were detected using One-way ANOVA (n=3).

Next, we validated that transfection with the hsa-miR-141-3p inhibitor significantly reduced hsa-miR-141-3p expression, whereas the hsa-miR-141-3p mimic markedly increased its levels (Fig 4A). Subsequently, we demonstrated that silencing hsa-miR-141-3p counteracted the effects of circFTO knockdown on HREC proliferation, migration, and tube formation (Fig 4B–4D). Furthermore, E-cadherin protein levels were decreased following hsa-miR-141-3p inhibitor treatment, while N-cadherin and Vimentin levels were upregulated (Fig 4E).

### hsa-miR-141-3p could bind to *ZEB1*

**Fig 5:**
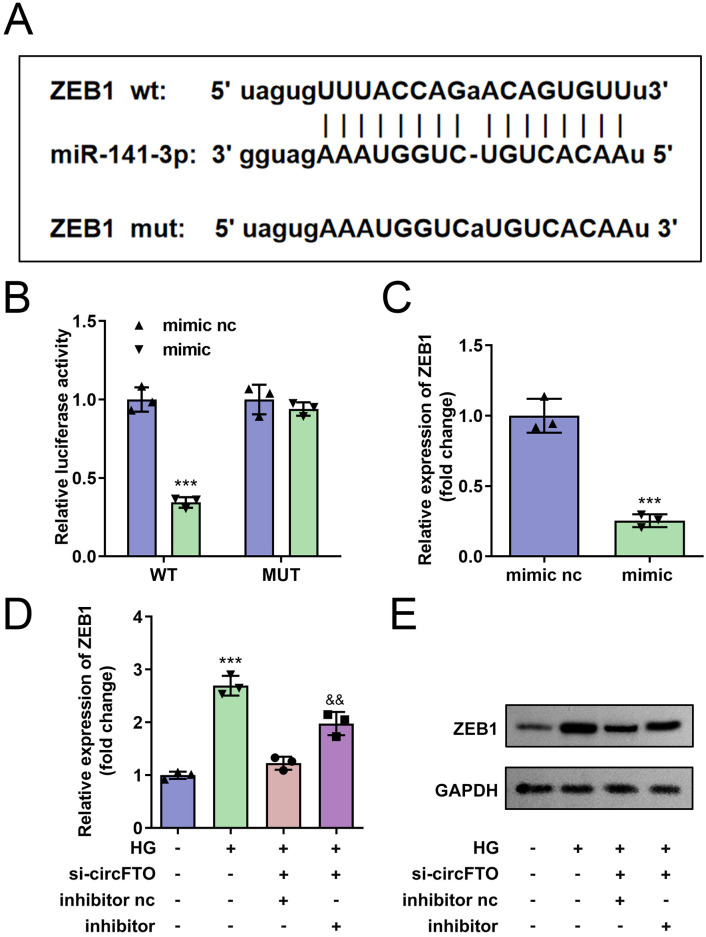
*hsa-miR-141-3p* targeted to *ZEB1.* A-B: The target *hsa-miR-141-3p* and binding sites of *ZEB1* was confirmed by the targetscan database and validated by the dual-luciferase reporter gene system. The *ZEB1* expressions in HRECs were measured with RT-qPCR after *hsa-miR-141-3p* mimic (C). The mRNA (D) and protein (E) levels of ZEB1 were detected by RT-qPCR and western blot in HG-treated and circFTO knockdown HRECs. (A-C) The differences were detected using Student-T test (n=3). (D) The differences were detected using One-way ANOVA (n=3).

The TargetScan database (http://www.targetscan.org/vert_72/) was used to identify potential target genes of hsa-miR-141-3p. Among these targets, ZEB1 is a key marker gene for epithelial-mesenchymal transition (EMT) progression and is closely associated with endothelial cell development, making it a focus for further experiments. The predicted binding sites between ZEB1 and hsa-miR-141-3p are shown in Fig 5A. Co-transfection of the ZEB1 3’UTR-WT construct with the hsa-miR-141-3p mimic significantly reduced luciferase activity compared to the mimic NC group (Fig 5B). Furthermore, overexpression of hsa-miR-141-3p markedly downregulated ZEB1 expression in HRECs (Fig 5C). The mRNA (Fig 5D) and protein (Fig 5E) levels of ZEB1 were upregulated in HG-treated HRECs, and circFTO knockdown significantly decreased the mRNA (Fig 5D) and protein (Fig 5E) levels of ZEB1 in HG-treated HRECs.

### Over expressed *ZEB1* abrogated the hsa-miR-141-3p mimic effects in HRECs

**Fig 6:**
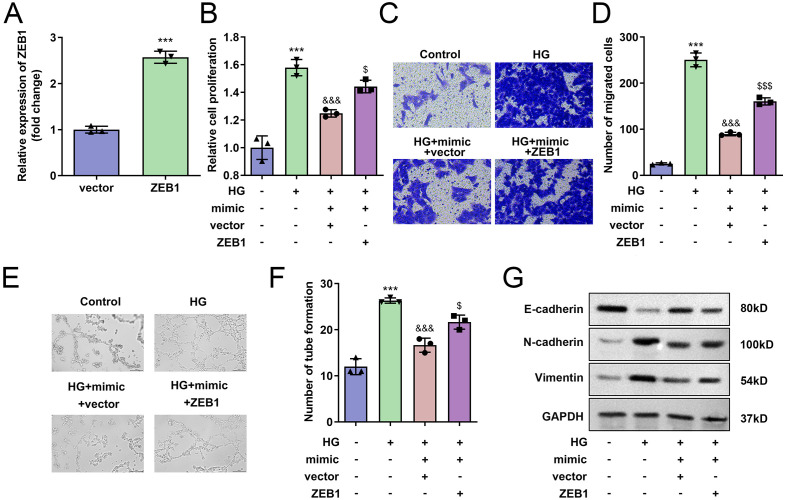
Overexpression of ZEB1 abrogated the hsa-miR-141-3p mimic effects in HRECs. A: Transfection efficiency of ZEB1. B: Cell proliferation was dected by MTT assay. C: The cell migration was detected with Transwell assay. D: Number of tube formation was determined. E: The protein levels of E-cadherin, N-cadherin and Vimentin were determined with western blot. **P<0.01, ***P<0.001 VS Control group. &P<0.05, &&P<0.01 VS HG group. $P<0.05 VS HG+hsa-miR-141-3p mimic +vector group. The differences were detected using One-way ANOVA (n=3).

Finally, we validated that ZEB1 expression was significantly upregulated following ZEB1 transfection (Fig 6A). Transfection with hsa-miR-141-3p mimic markedly suppressed HREC proliferation, migration, and tube formation compared to controls (Fig 6B–6D), an effect that was reversed upon ZEB1 overexpression. Furthermore, ZEB1 upregulation counteracted the regulatory effects of hsa-miR-141-3p mimic on E-cadherin, N-cadherin, and Vimentin expression levels in HRECs (Fig 6E).

## Discussion

In the current study, we confirmed that circFTO was significantly upregulated in PDR patients as well as in HRECs stimulated by HG. Interestingly, circFTO knockdown significantly inhibited HREC proliferation, migration, and tube formation. Furthermore, we demonstrated that circFTO mediates PDR progression by modulating the hsa-miR-141-3p/ZEB1 axis.

EMT is a critical process in cell proliferation and migration [37]. As both a physiological and pathological phenomenon, EMT involves the loss of epithelial cell characteristics (e.g., reduced E-cadherin expression) and the acquisition of mesenchymal traits, such as increased expression of N-cadherin and Vimentin [38,39]. This transition is characterized by the downregulation of E-cadherin, a membrane protein that mediates adherens junctions between epithelial cells, and the upregulation of mesenchymal markers like N-cadherin and Vimentin, which facilitate interactions between stromal cells. Excessive proliferation and migration are hallmark features of PDR [40]. Yang et al. [41] demonstrated that E-cadherin was downregulated, while N-cadherin and Vimentin were upregulated in HG-treated ARPE-19 cells, indicating that EMT plays a central role in DR progression. Similarly, in this study, we observed reduced E-cadherin protein levels and increased N-cadherin and Vimentin expression in HG-stimulated HRECs. These findings suggest that modulating EMT progression may represent a novel therapeutic strategy for PDR.

In recent years, numerous studies have demonstrated that circRNAs are single-stranded, covalently closed circular RNAs lacking 5’ and 3’ terminal structures [18]. CircRNAs are broadly classified into three categories: exonic circRNAs (ecircRNAs), intronic circRNAs (ciRNAs), and exon-intron circRNAs (EIciRNAs) [42]. Accumulating evidence suggests that various circRNAs play critical roles in the pathogenesis of DR. For instance, Li et al. [43] reported that circRNA_0084043 was upregulated in HG-treated ARPE-19 cells, contributing to oxidative stress and inflammatory responses in DR development. Jiang et al. [44] further demonstrated that circZNF532 modulates pericyte proliferation and differentiation in DR. Similarly, our current study revealed that circFTO was significantly elevated in both PDR tissues and HG-treated HRECs. Knockdown of circFTO suppressed the biological behaviors (e.g., proliferation, migration, and tube formation) of HG-stimulated HRECs. These findings align with those of He et al. [24], further supporting the involvement of circRNAs in DR progression..

circRNAs, functioning as sponge adsorbents or ceRNA molecules, compete with miRNAs to regulate mRNA expression [22]. MiRNAs are well-established inhibitors of mRNA translation and play critical roles in modulating diverse biological processes [45]. Accumulating evidence indicates that circRNAs regulate cellular behavior in DR by sponging miRNAs. For example, Zou et al. [46] demonstrated that circCOL1A2 promotes DR progression by negatively regulating miR-29b expression. Similarly, Li et al. [43] showed that circRNA_0084043 alleviates pathological DR development by sponging miR-140-3p. In this study, we identified that circFTO acts as a sponge for hsa-miR-141-3p in high-glucose (HG)-treated HRECs using bioinformatic tools. Dual luciferase reporter and RNA-pull down assays further validated that circFTO directly interacts with hsa-miR-141-3p, which has been shown to inhibit proliferation and migration in various diseases. Zhang et al. [47] reported that hsa-miR-141-3p suppresses vascular smooth muscle cell growth and migration, mitigating atherosclerosis by targeting the Keap1/Nrf2/HO-1 signaling pathway. Additionally, hsa-miR-141-3p exhibits anti-proliferative effects in cancers, including colorectal cancer [48], osteosarcoma [49], and breast cancer [50]. In our research, hsa-miR-141-3p levels were elevated in HG-treated HRECs, and its inhibition counteracted the effects of circFTO knockdown. Zhang et al. [30] further demonstrated that hsa-miR-141-3p suppresses retinal vascular endothelial cell growth while promoting retinal ganglion cell apoptosis, findings consistent with our results. Collectively, these data indicate that circFTO drives PDR progression by sponging hsa-miR-141-3p.

Subsequently, we confirmed that circFTO upregulates ZEB1 expression through competitive binding with hsa-miR-141-3p. As a transcriptional repressor of E-cadherin during epithelial-mesenchymal transition, ZEB1 suppression leads to diminished E-cadherin levels [51]. For instance, miR-873 modulates papillary thyroid cancer progression by regulating ZEB1 expression [52]. Additionally, Han et al. [53] demonstrated that miRNA-150-5p negatively regulates ZEB1 in diabetic cardiomyopathy development. However, the role of ZEB1 in PDR remains largely unexplored. In our study, hsa-miR-141-3p directly targets ZEB1 mRNA. Overexpression of hsa-miR-141-3p significantly reduced ZEB1 expression, whereas enforced ZEB1 overexpression reversed the effects of hsa-miR-141-3p mimic in HERCs. These findings suggest that circFTO promotes hyperglycemia-induced HERC biological behaviors through modulation of the hsa-miR-141-3p/ZEB1 axis.

Notably, the observed elevation of circFTO levels in PDR patient cohorts provides preliminary evidence supporting its potential as a diagnostic biomarker, which warrants further investigation in larger clinical studies. However, translating these findings into therapeutic applications faces significant challenges, particularly regarding the development of safe and effective methods to inhibit circFTO specifically in the retinal tissue. Future studies utilizing preclinical models are essential to validate the therapeutic potential of targeting the circFTO/hsa-miR-141-3p/ZEB1 axis and to explore its interplay with established PDR pathways (e.g., VEGF signaling). However, there were still some limitations in this study. For the detection of circFTO expression in clinical practice, we were unable to collect normal retinal fibrovascular membranes. So the aqueous humor samples from cataract patients were used as controls. We will plan to conduct more clinical trials in the future to address this issue.

In summary, circFTO acts as a molecular sponge for hsa-miR-141-3p in PDR by regulating ZEB1 expression. Moreover, circFTO may modulate the biological behavior of HERCs via the hsa-miR-141-3p/ZEB1 regulatory axis. These findings establish a novel conceptual framework for understanding PDR pathogenesis, offering mechanistic insights into circRNA-mediated microvascular dysfunction. They highlight the circFTO/hsa-miR-141-3p/ZEB1 axis as a potential target for further investigation, though its therapeutic applicability remains to be established through future preclinical and clinical studies.

## Supporting information

S1 Data Original Data. (ZIP)

S2 Data Raw data of WB. (ZIP)
