# Supplementary figures and images for: Circular RNA FTO functions as a hsa-miR-141-3p sponge to regulate the growth and migration abilities of human retinal endothelial cells via up-regulating ZEB1
Source: PLoS One. 2025 Dec 5;20(12):e0338208. doi: 10.1371/journal.pone.0338208 (PMC12680237; doi:10.1371/journal.pone.0338208)

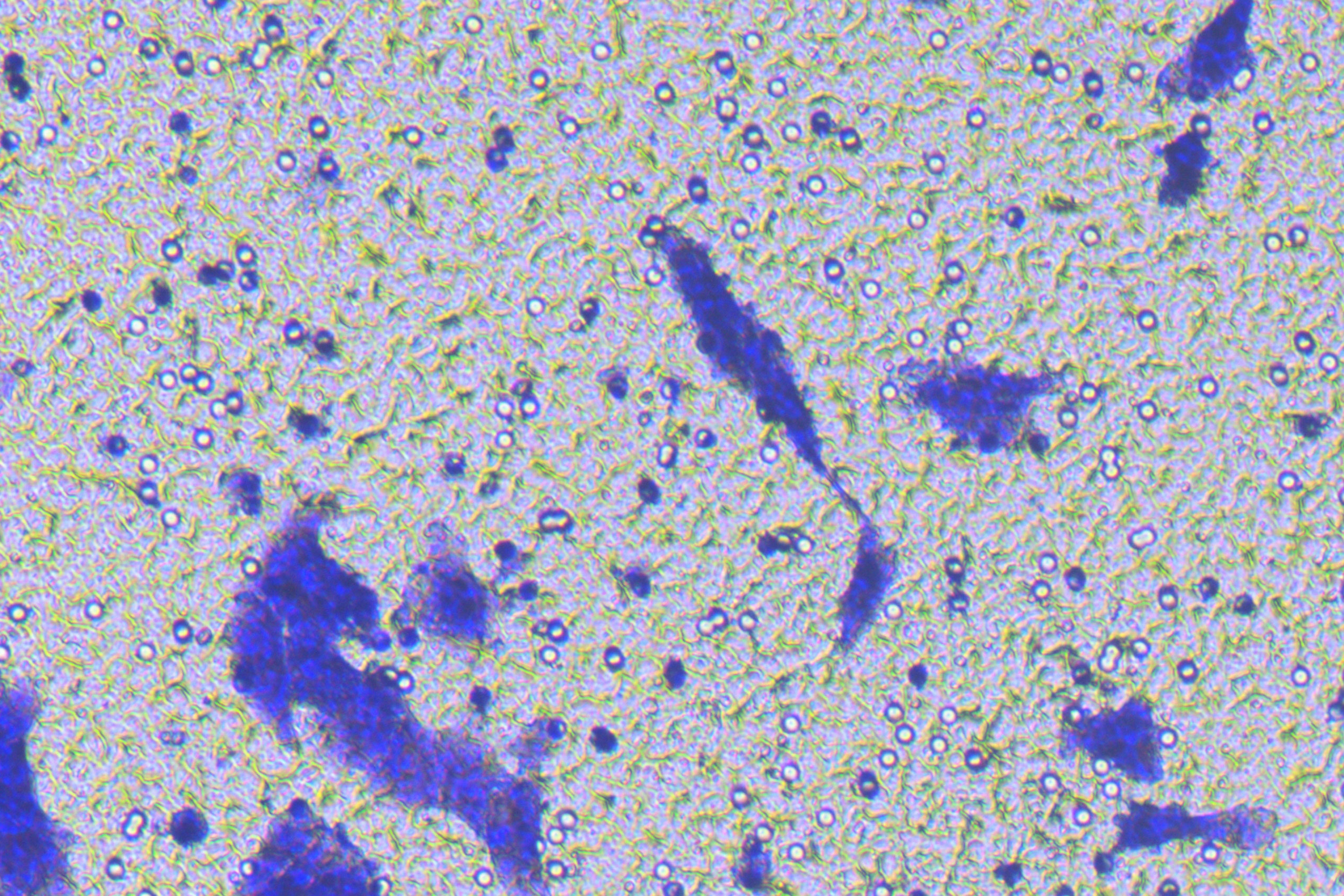

Supplement: S1 Data — (ZIP) [file pone.0338208.s001.zip › YT2021040602-original data/2C/2-1 (1).jpg]

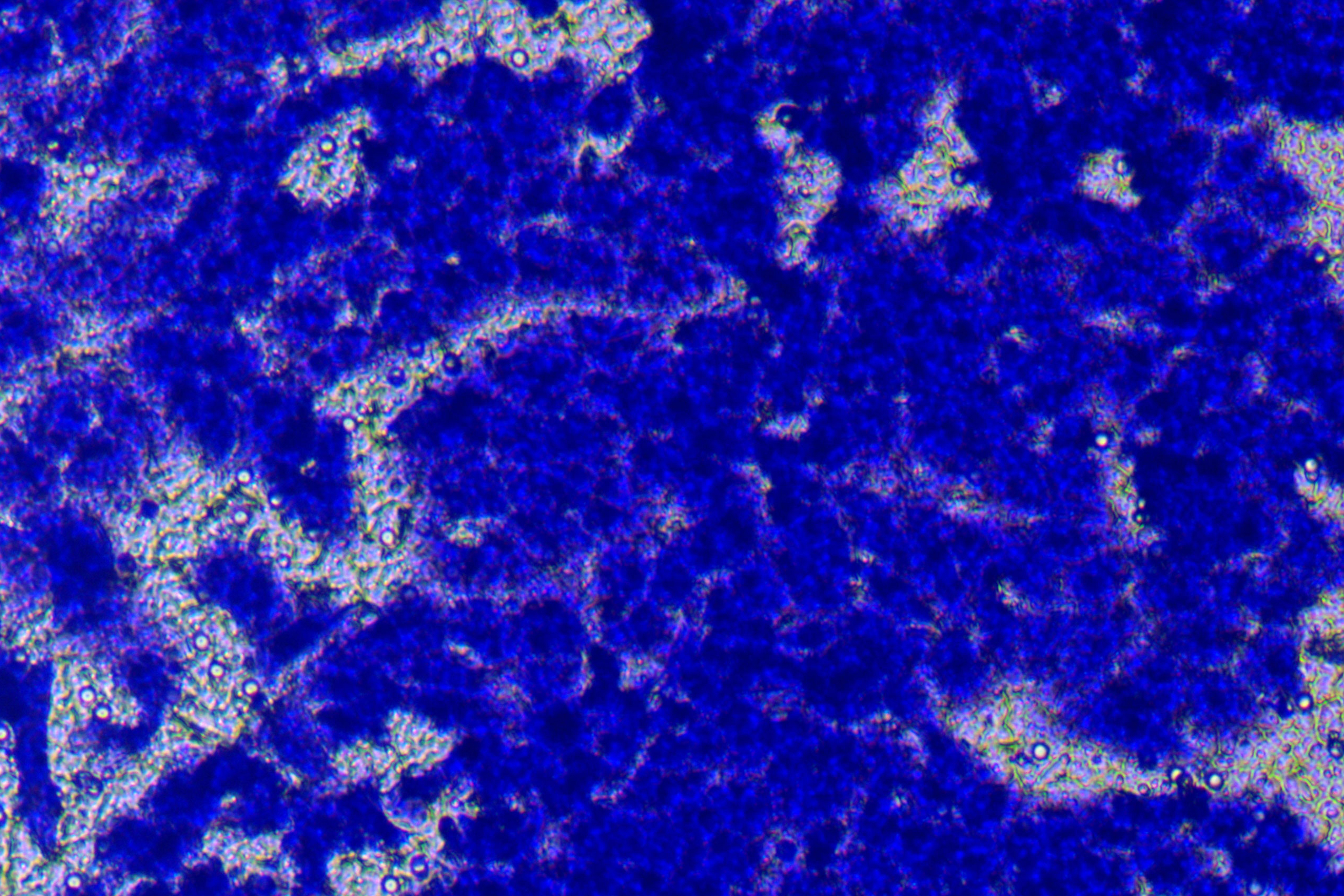

Supplement: S1 Data — (ZIP) [file pone.0338208.s001.zip › YT2021040602-original data/2C/2-1 (2).jpg]

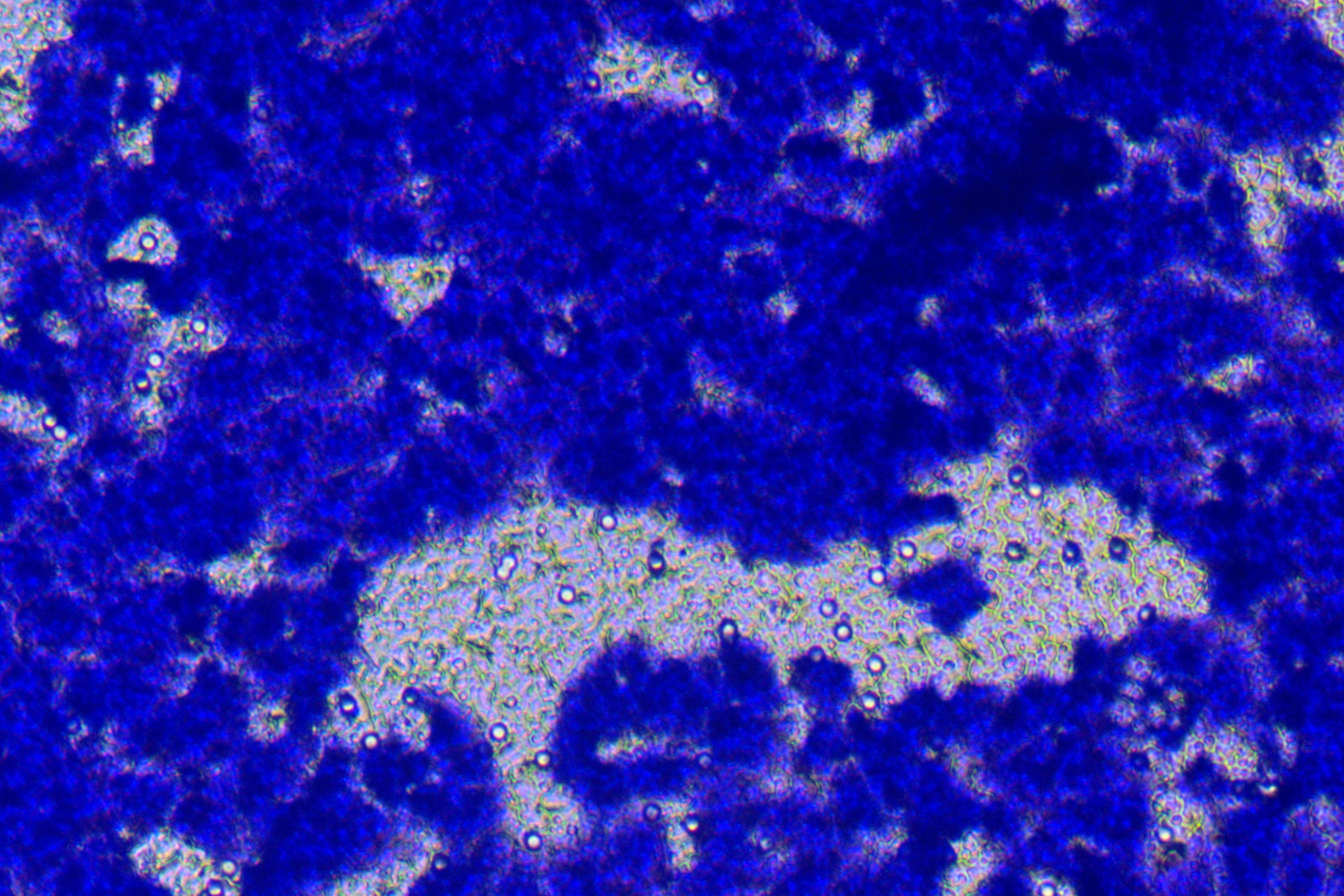

Supplement: S1 Data — (ZIP) [file pone.0338208.s001.zip › YT2021040602-original data/2C/2-1 (3).jpg]

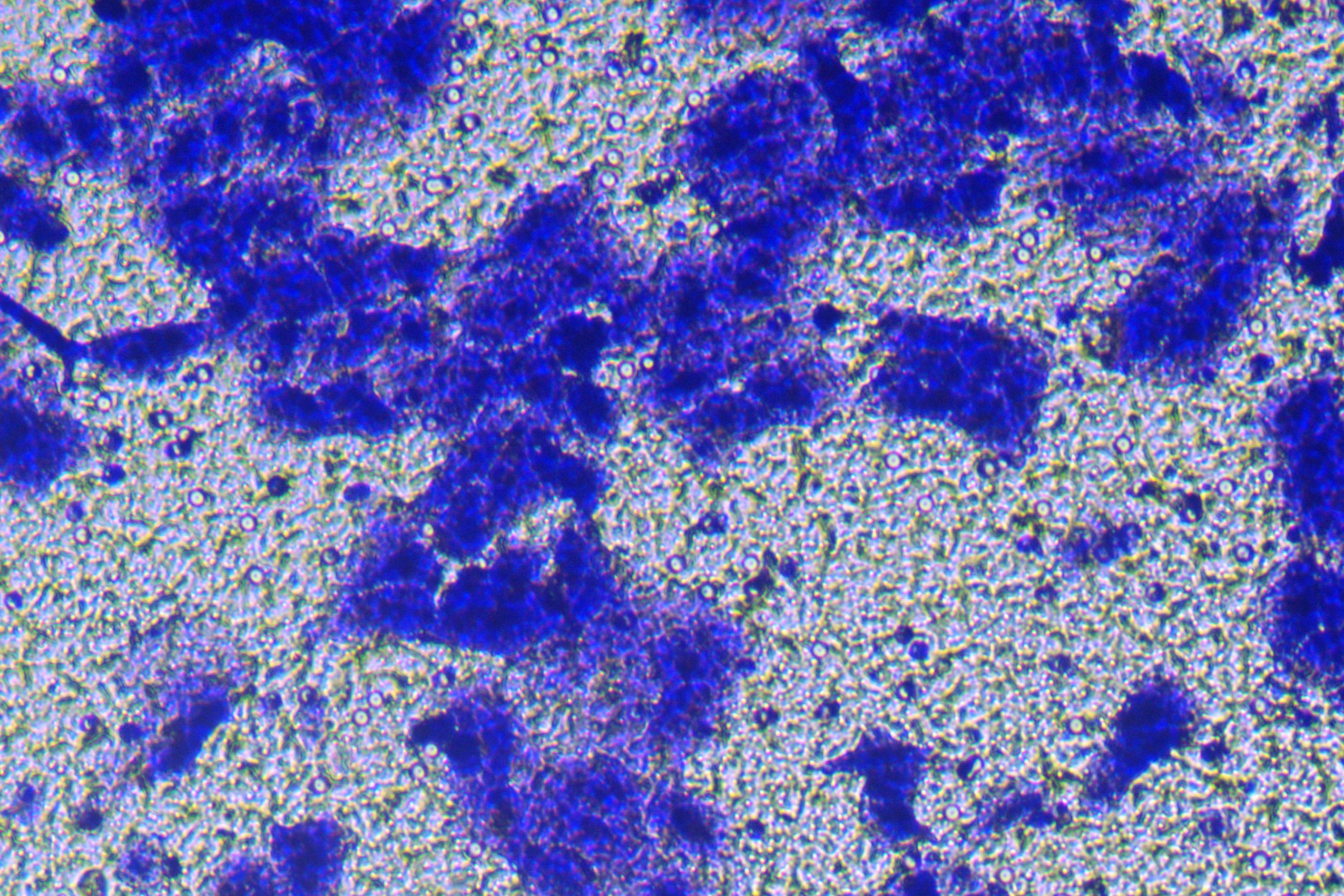

Supplement: S1 Data — (ZIP) [file pone.0338208.s001.zip › YT2021040602-original data/2C/2-1 (4).jpg]

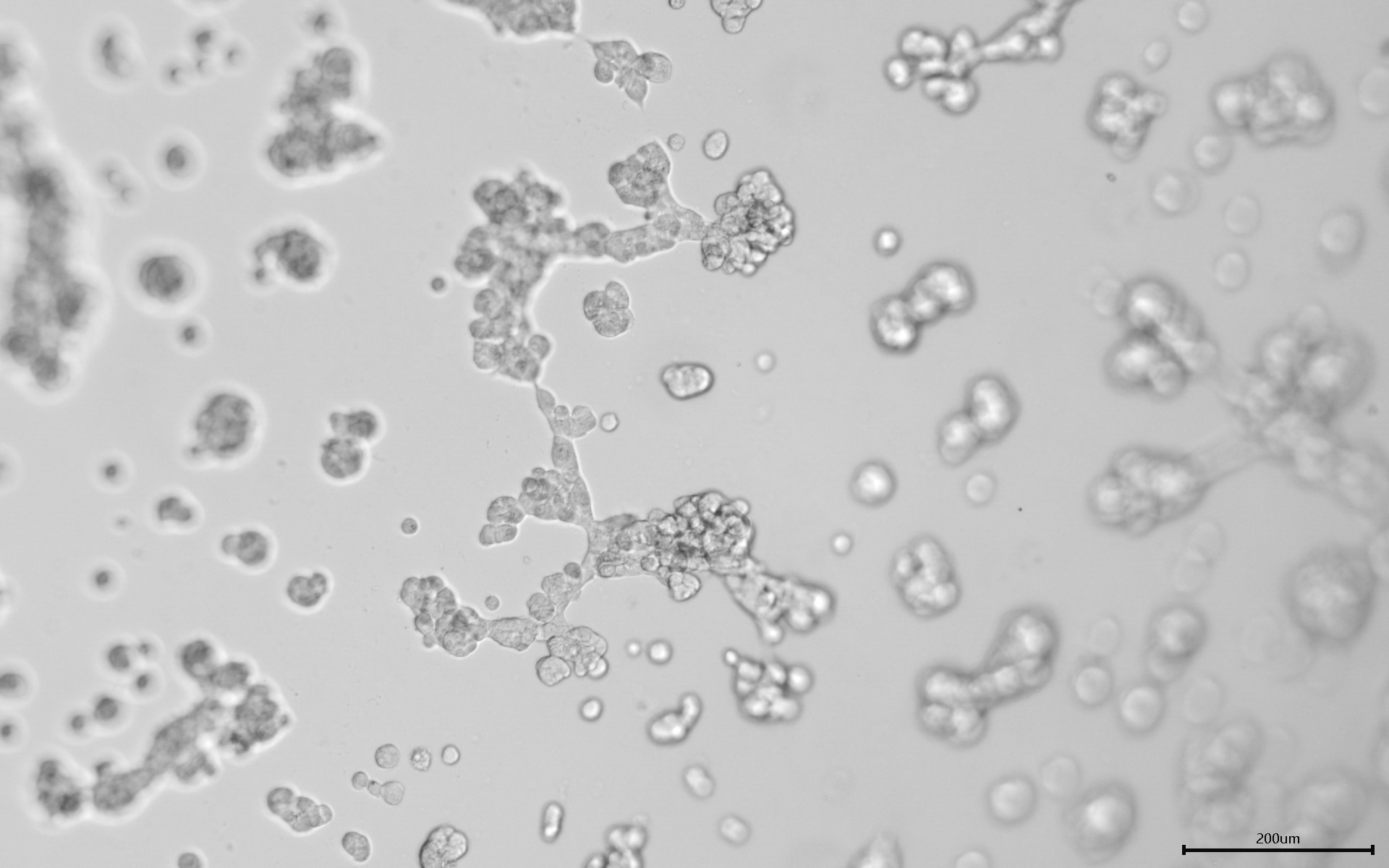

Supplement: S1 Data — (ZIP) [file pone.0338208.s001.zip › YT2021040602-original data/2E/2-1.jpg]

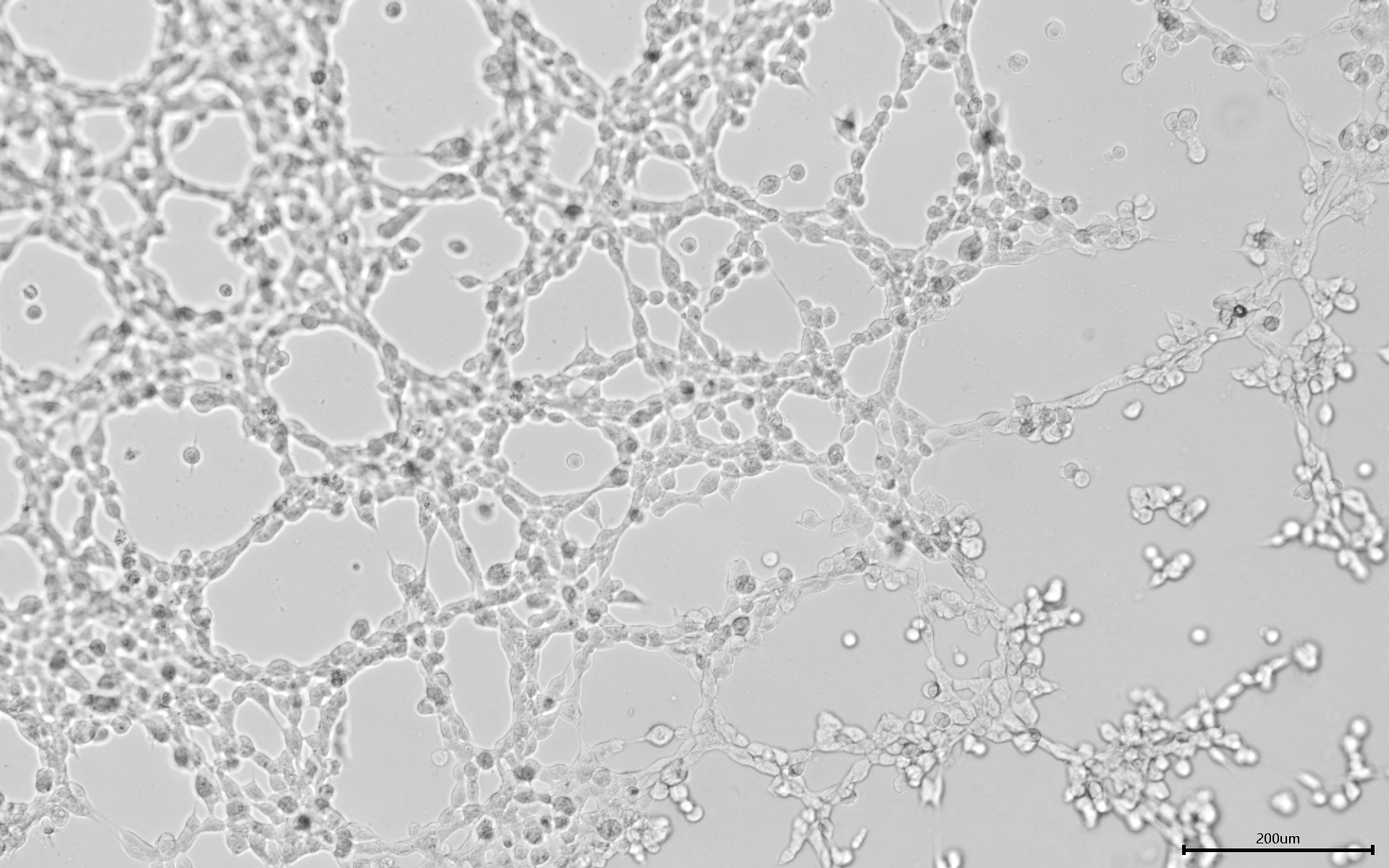

Supplement: S1 Data — (ZIP) [file pone.0338208.s001.zip › YT2021040602-original data/2E/2-2.jpg]

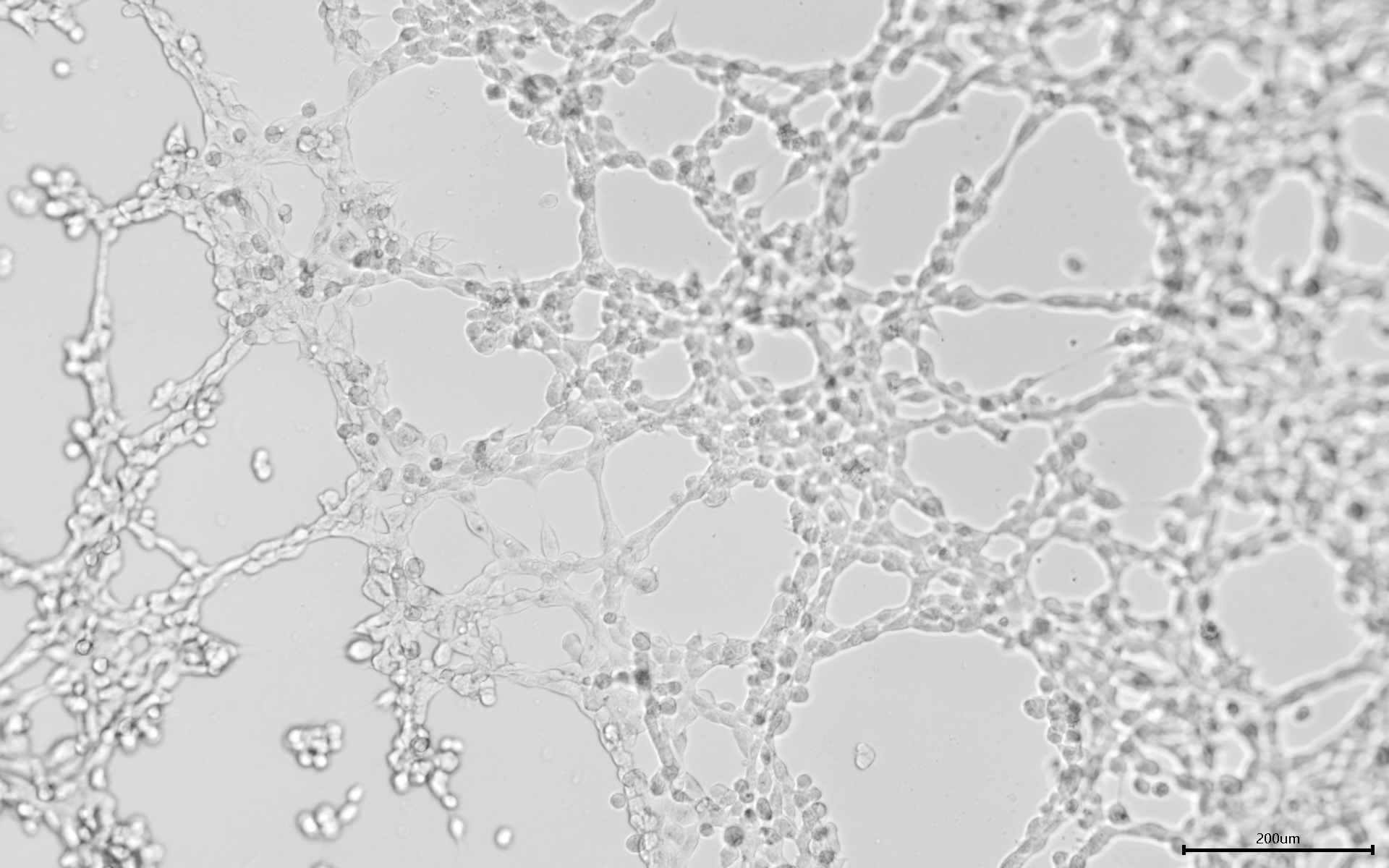

Supplement: S1 Data — (ZIP) [file pone.0338208.s001.zip › YT2021040602-original data/2E/2-3.jpg]

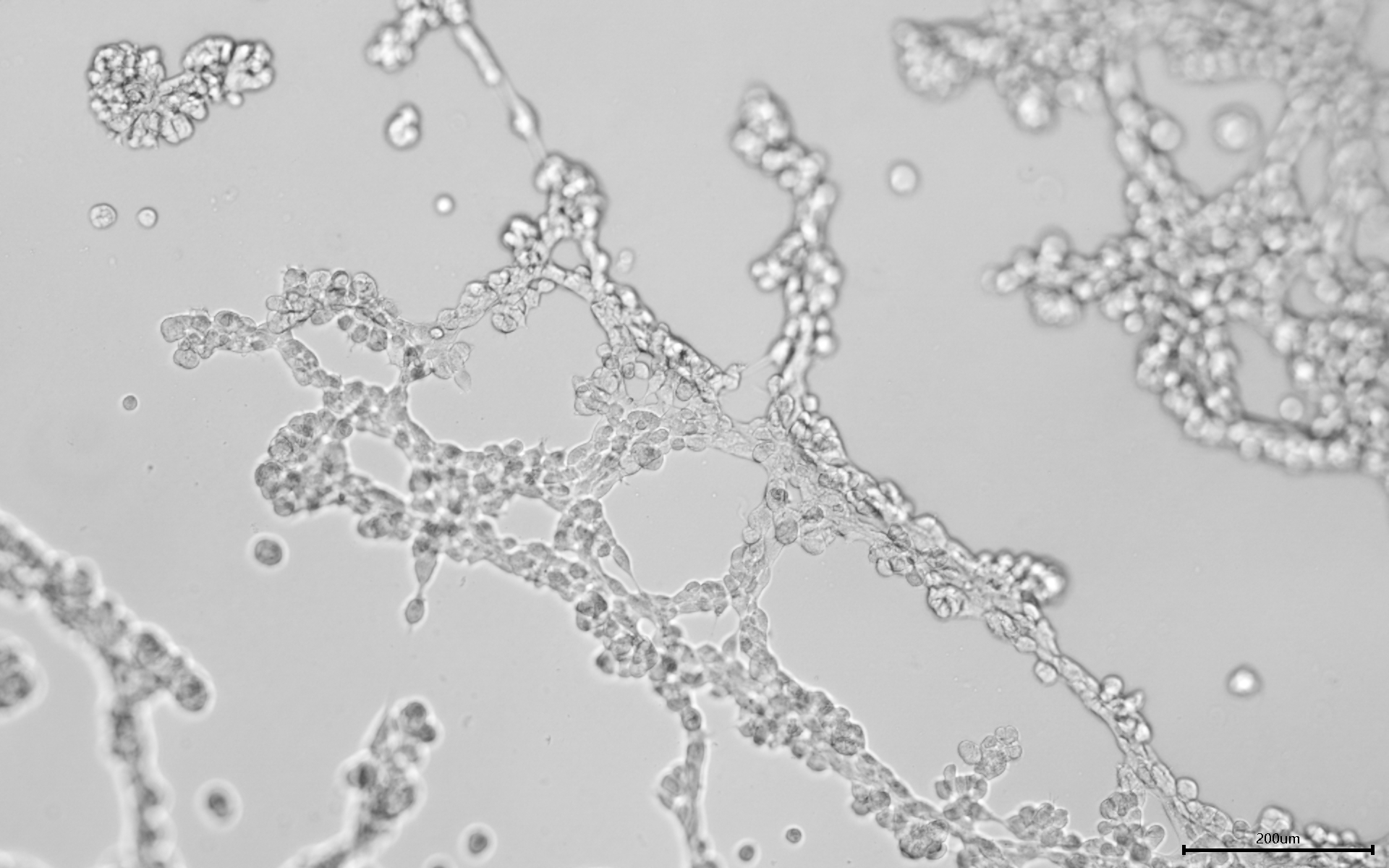

Supplement: S1 Data — (ZIP) [file pone.0338208.s001.zip › YT2021040602-original data/2E/2-4.jpg]

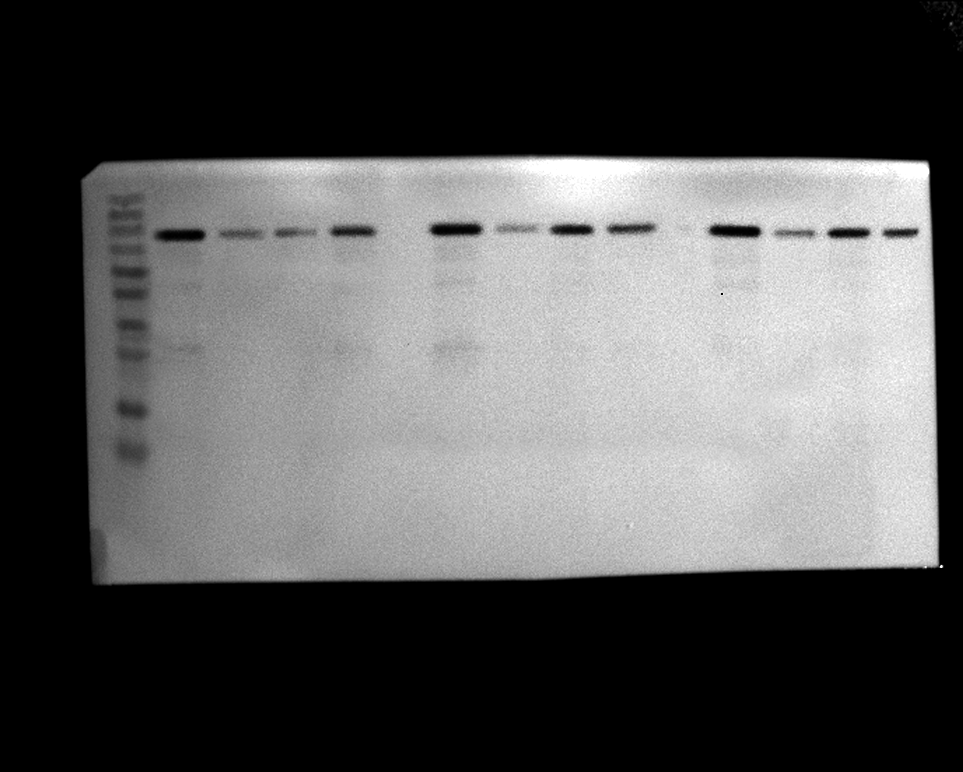

Supplement: S1 Data — (ZIP) [file pone.0338208.s001.zip › YT2021040602-original data/2G+4G+6G/1-E-cadherin.tif]

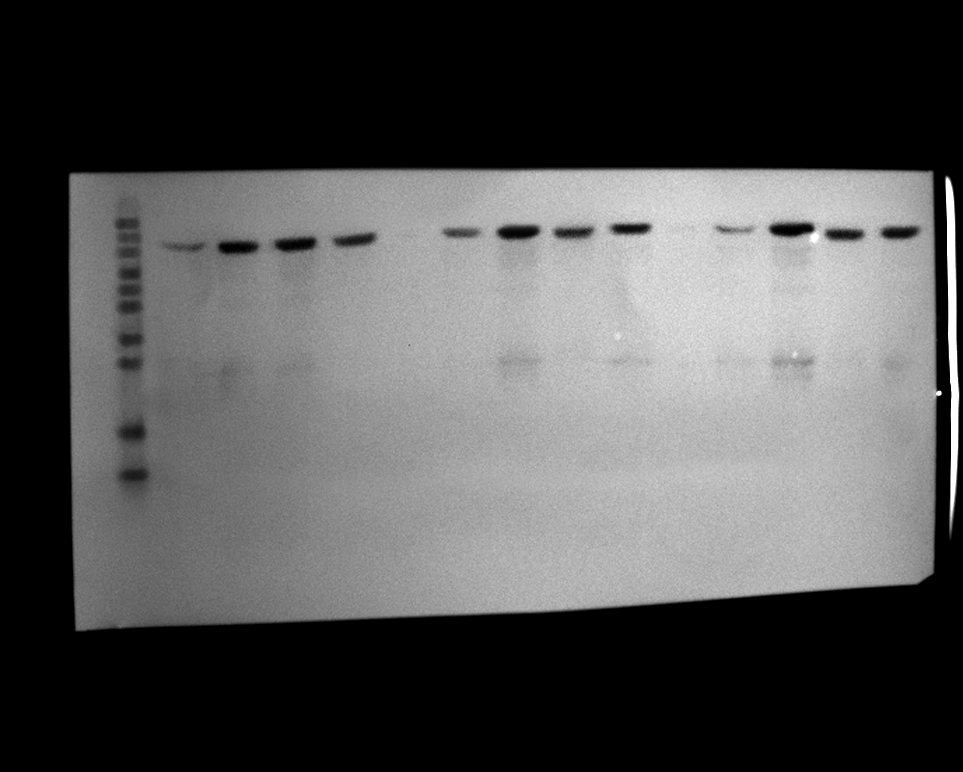

Supplement: S1 Data — (ZIP) [file pone.0338208.s001.zip › YT2021040602-original data/2G+4G+6G/2-N-cadherin.tif]

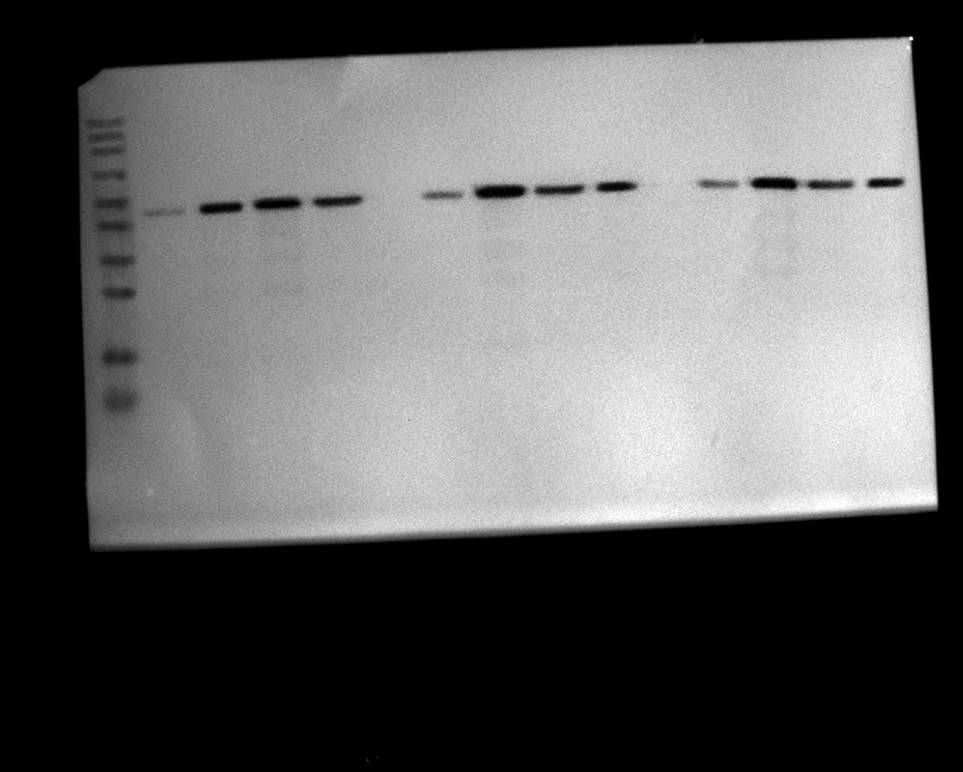

Supplement: S1 Data — (ZIP) [file pone.0338208.s001.zip › YT2021040602-original data/2G+4G+6G/3-Vimentin.tif]

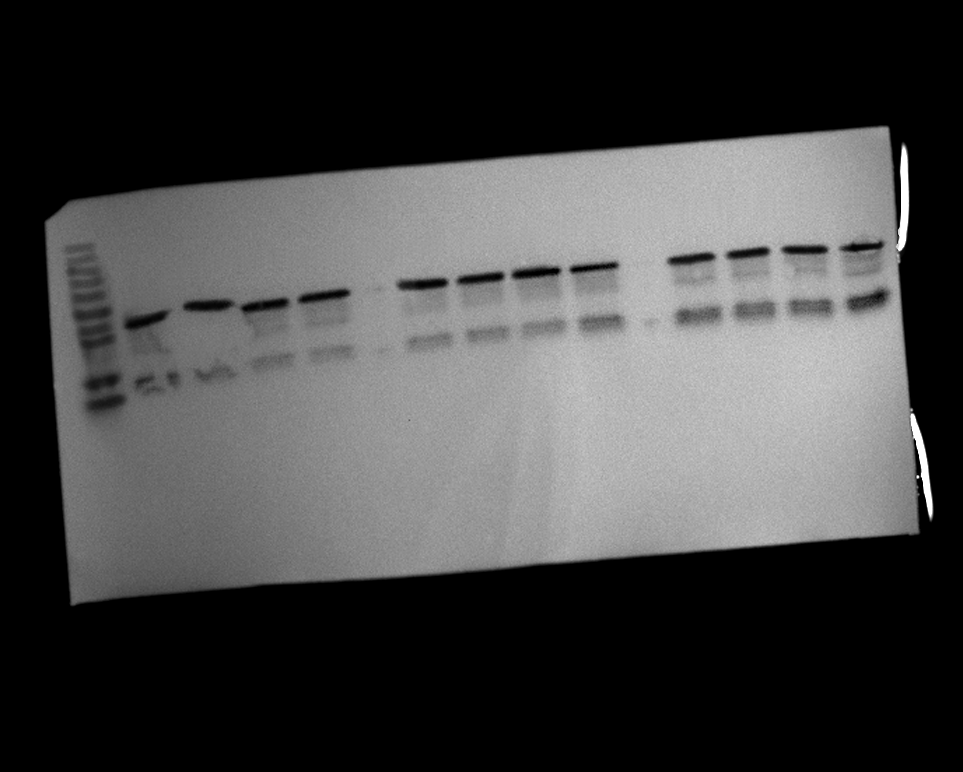

Supplement: S1 Data — (ZIP) [file pone.0338208.s001.zip › YT2021040602-original data/2G+4G+6G/4-GAPDH.tif]

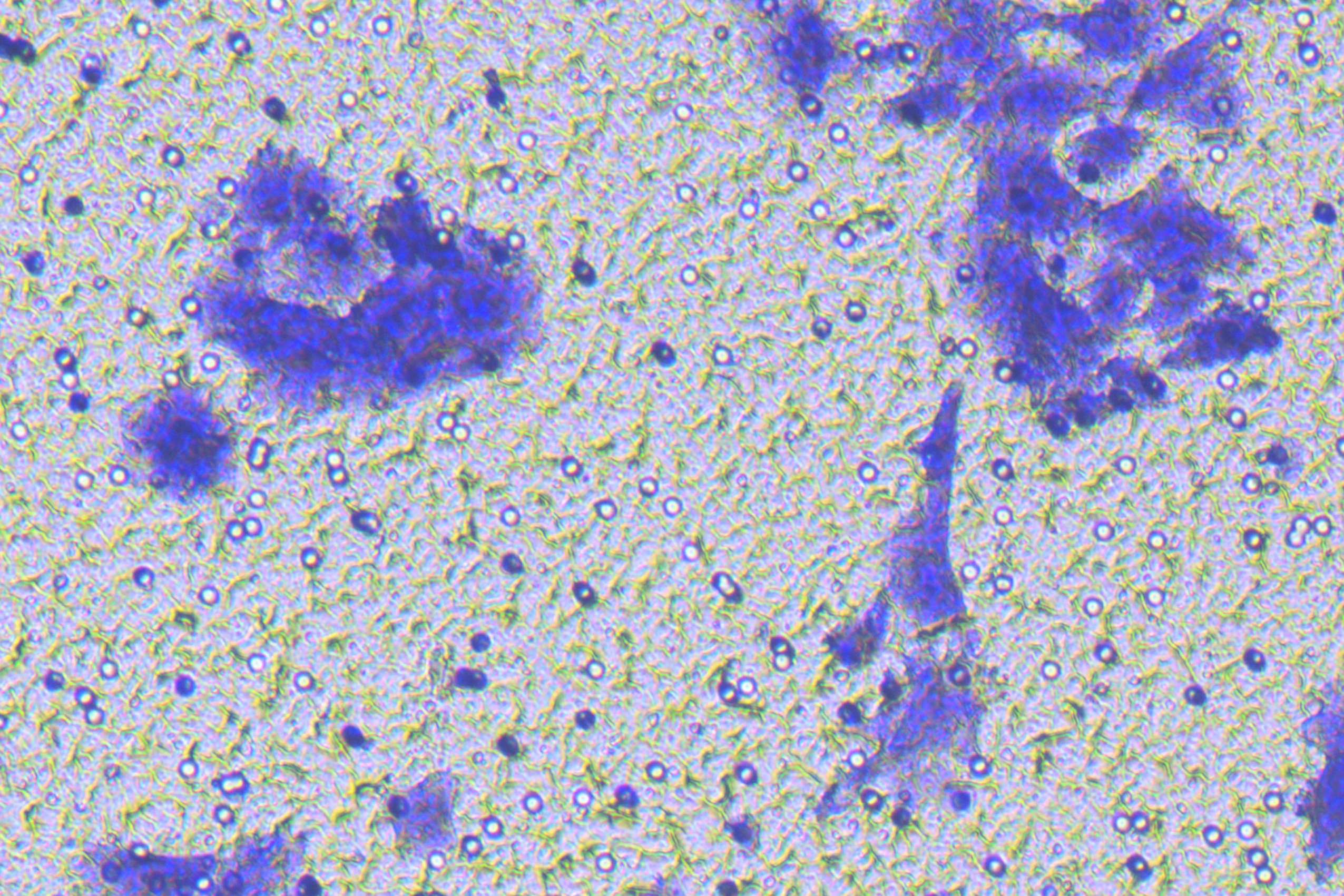

Supplement: S1 Data — (ZIP) [file pone.0338208.s001.zip › YT2021040602-original data/4C/4-1 (1).jpg]

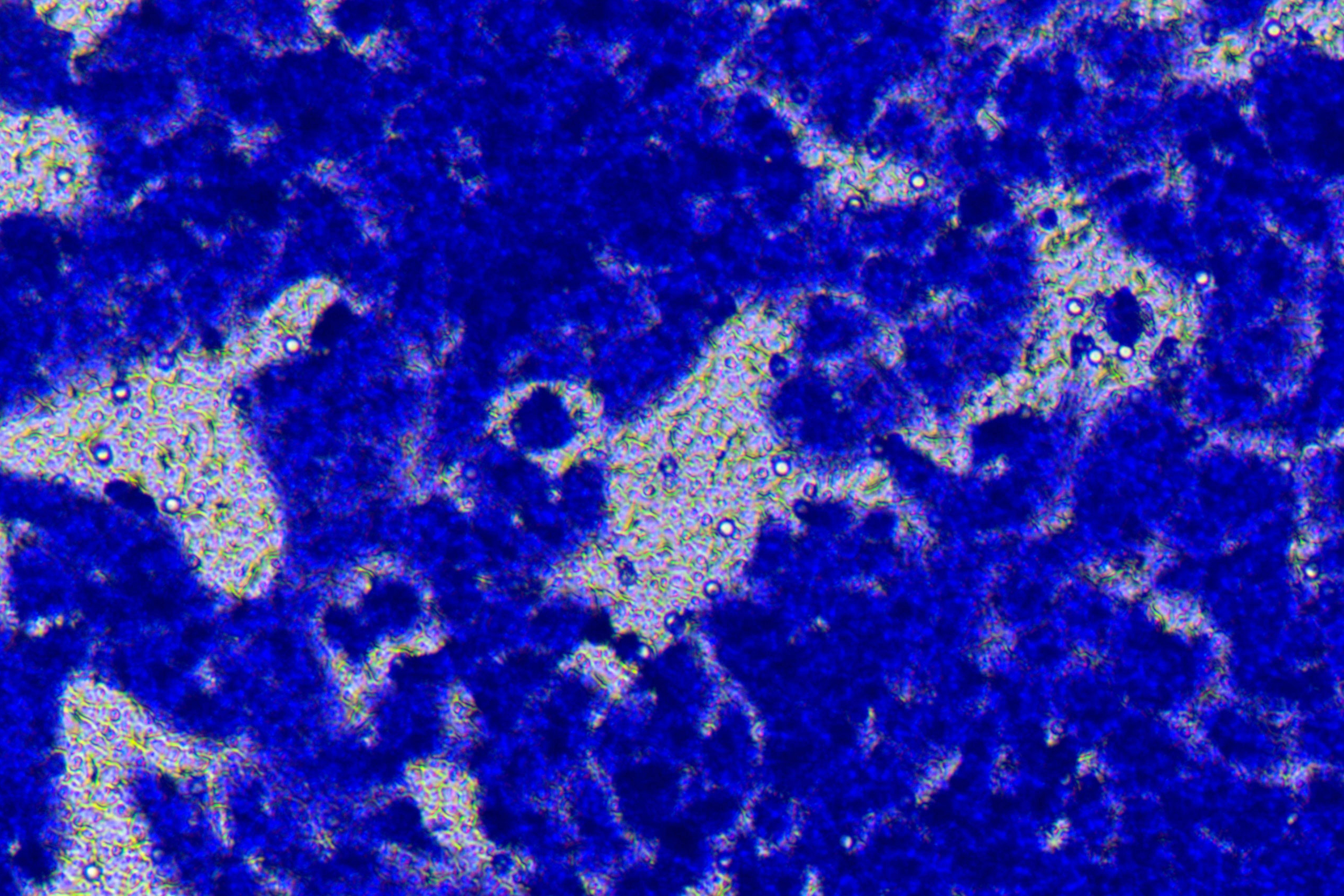

Supplement: S1 Data — (ZIP) [file pone.0338208.s001.zip › YT2021040602-original data/4C/4-1 (2).jpg]

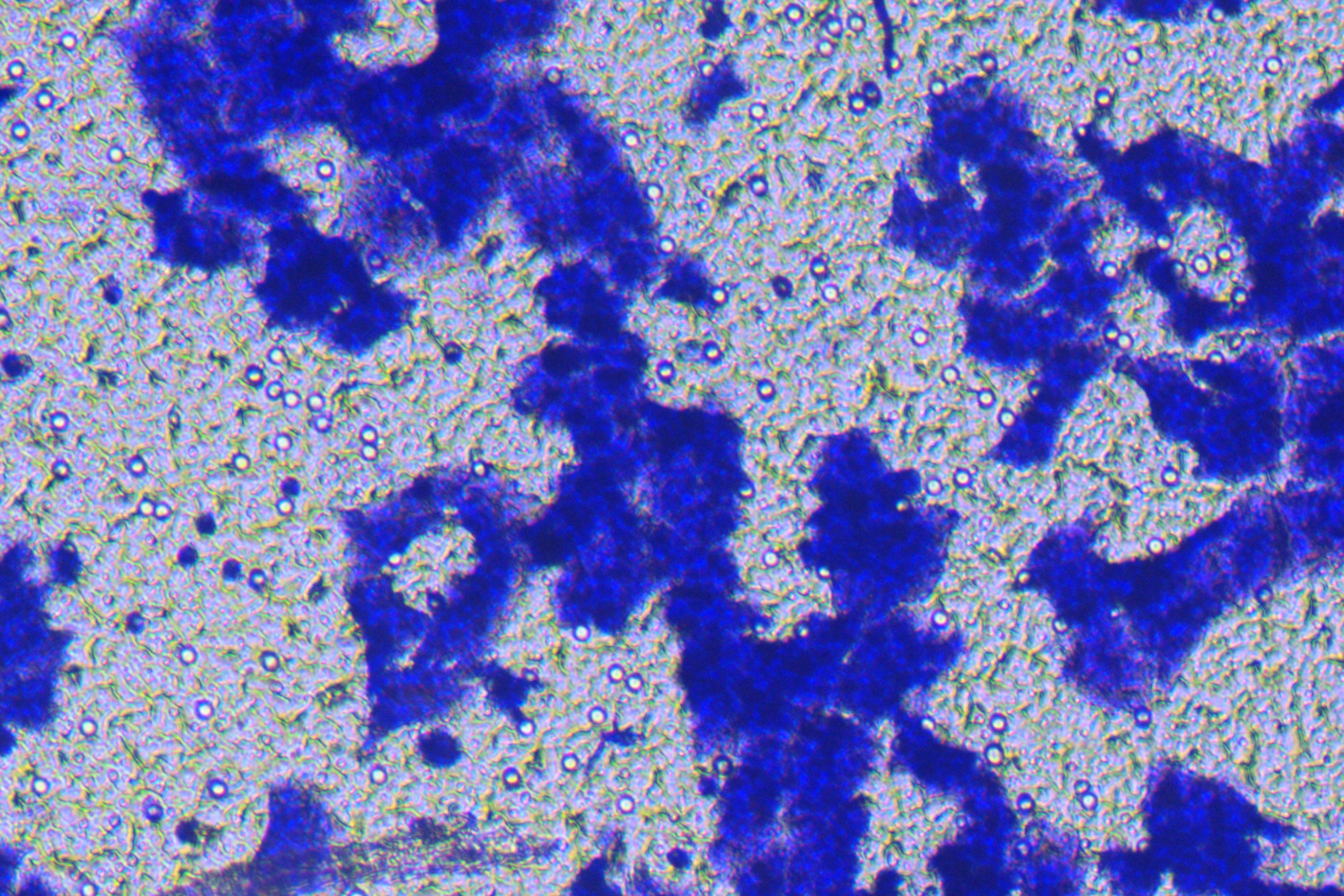

Supplement: S1 Data — (ZIP) [file pone.0338208.s001.zip › YT2021040602-original data/4C/4-1 (3).jpg]

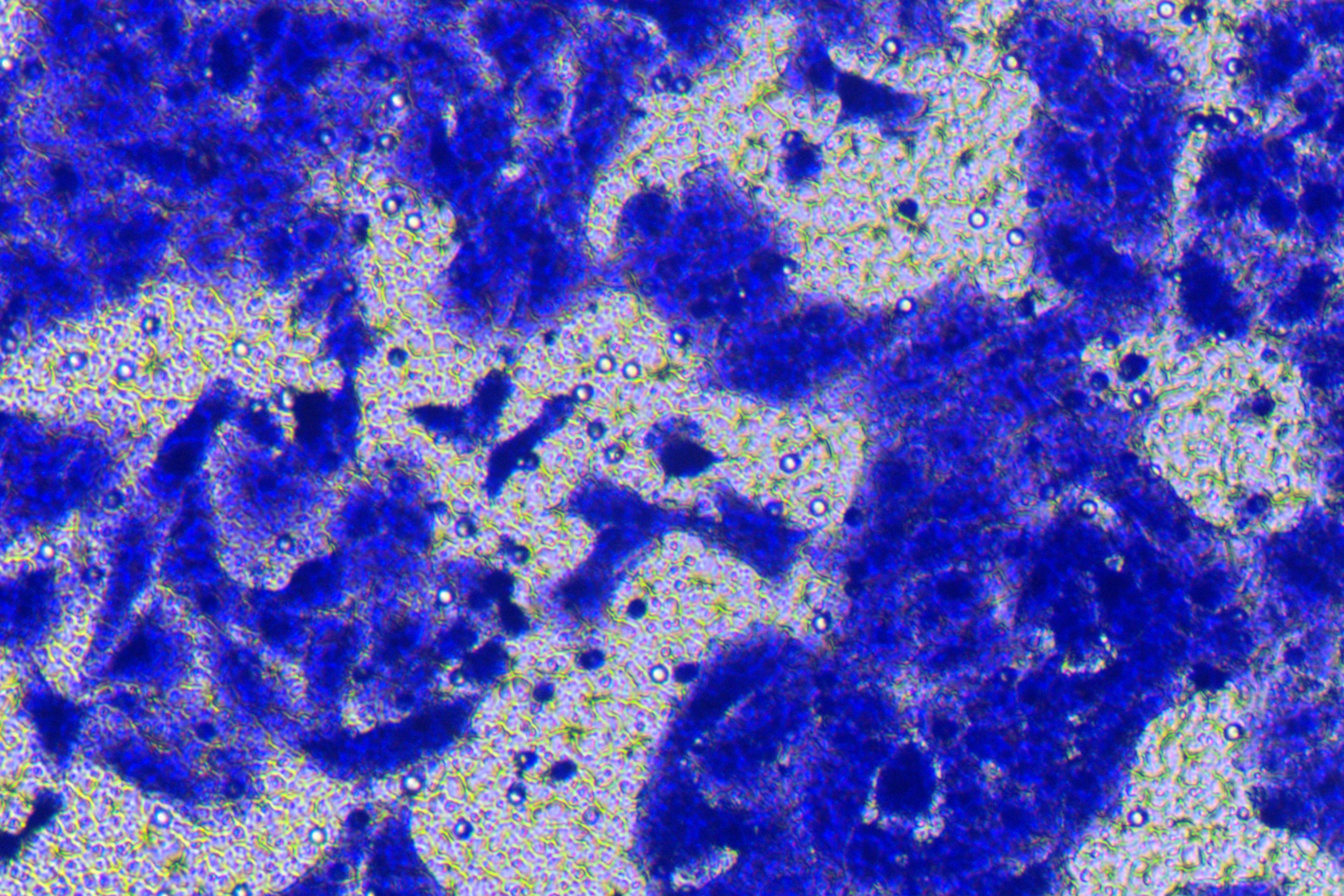

Supplement: S1 Data — (ZIP) [file pone.0338208.s001.zip › YT2021040602-original data/4C/4-1 (4).jpg]

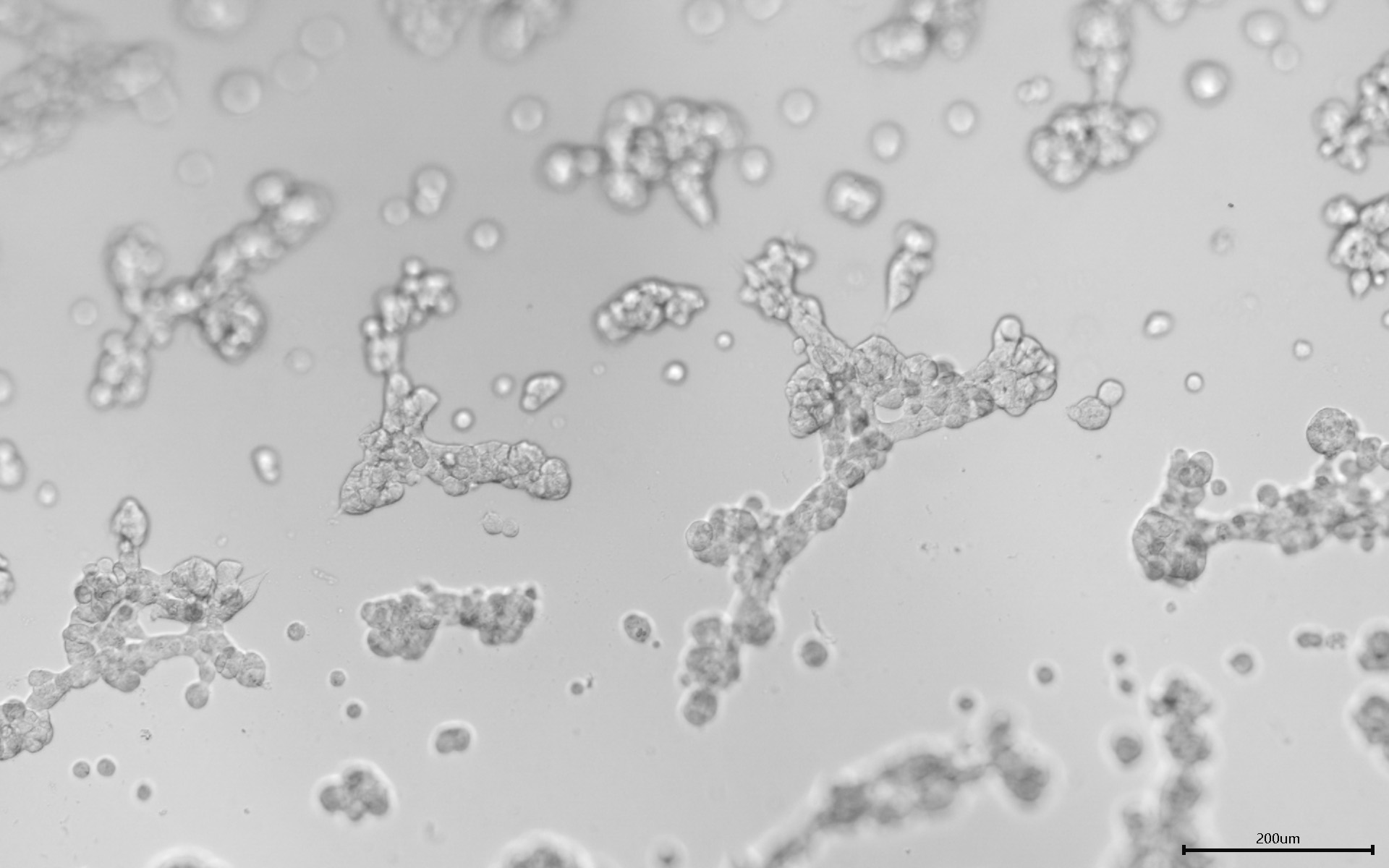

Supplement: S1 Data — (ZIP) [file pone.0338208.s001.zip › YT2021040602-original data/4E/4-1.jpg]

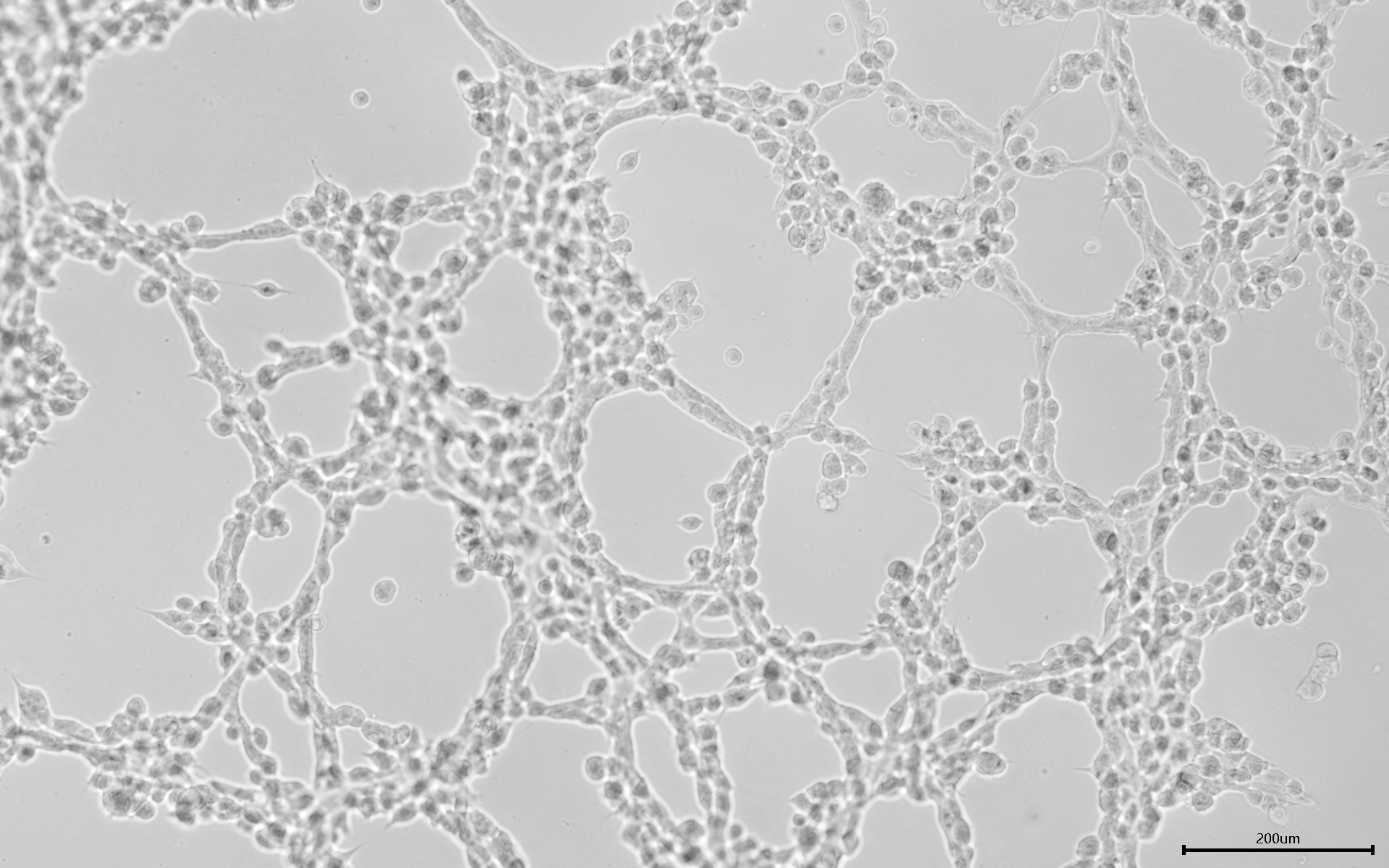

Supplement: S1 Data — (ZIP) [file pone.0338208.s001.zip › YT2021040602-original data/4E/4-2.jpg]

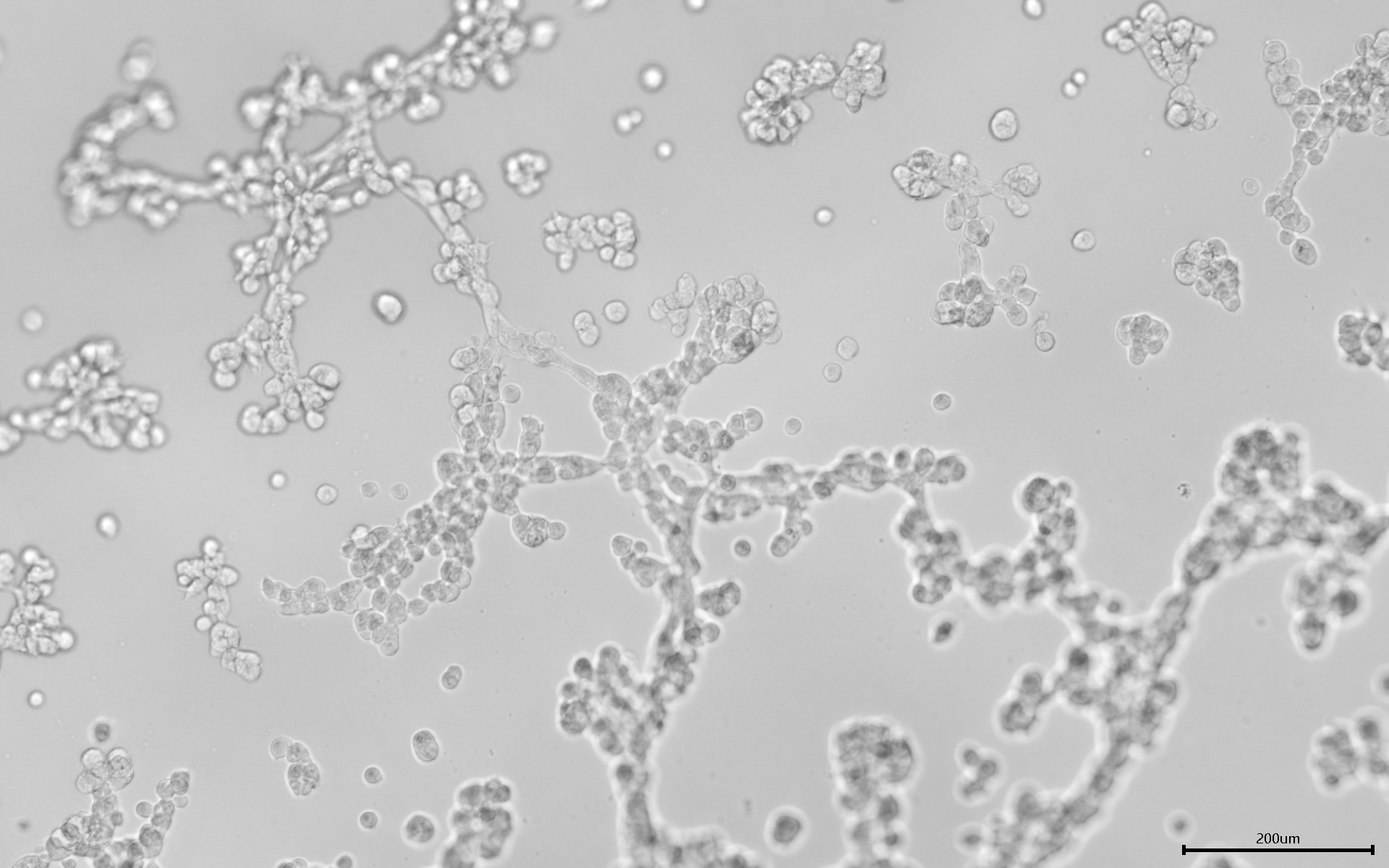

Supplement: S1 Data — (ZIP) [file pone.0338208.s001.zip › YT2021040602-original data/4E/4-3.jpg]

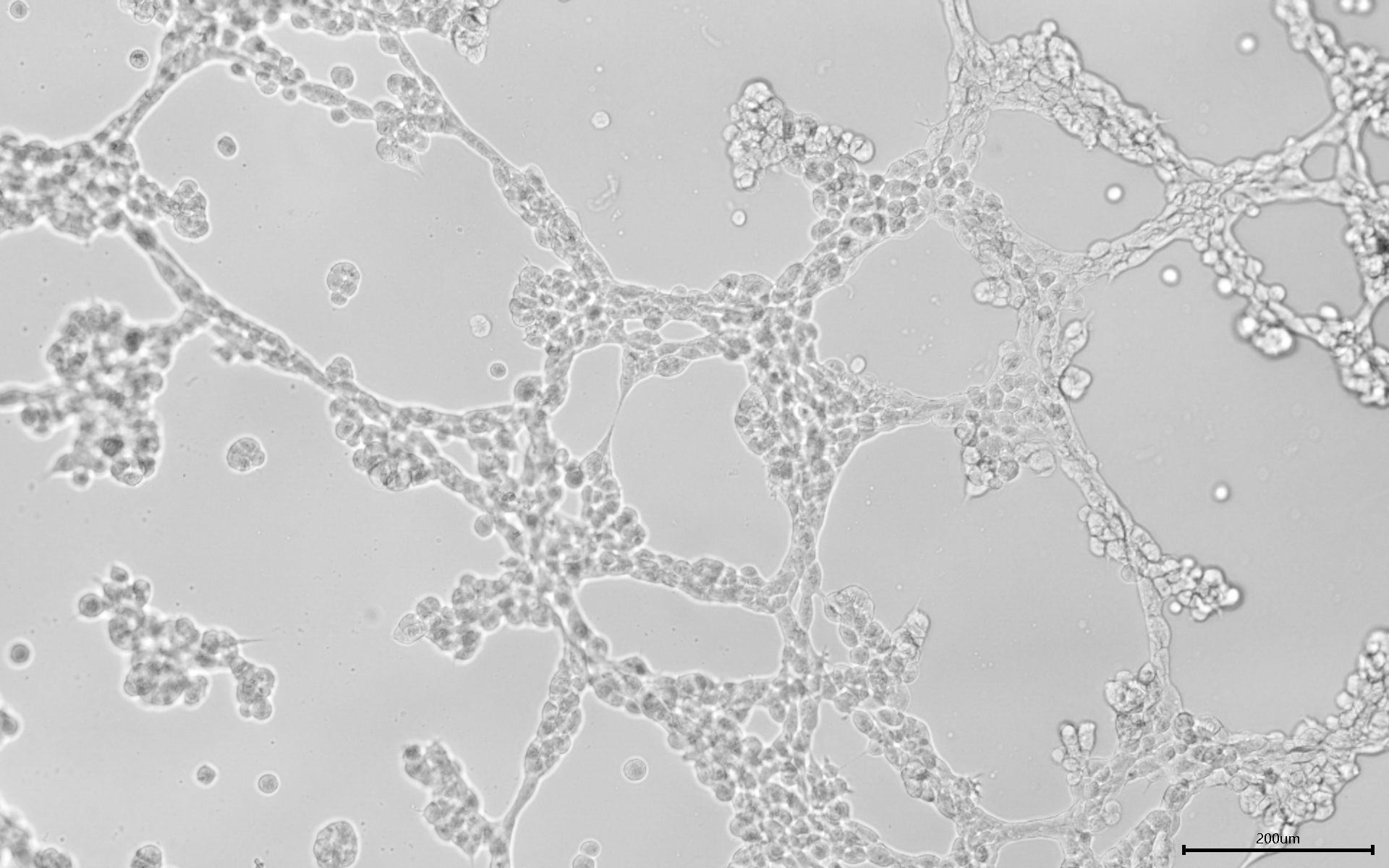

Supplement: S1 Data — (ZIP) [file pone.0338208.s001.zip › YT2021040602-original data/4E/4-4.jpg]

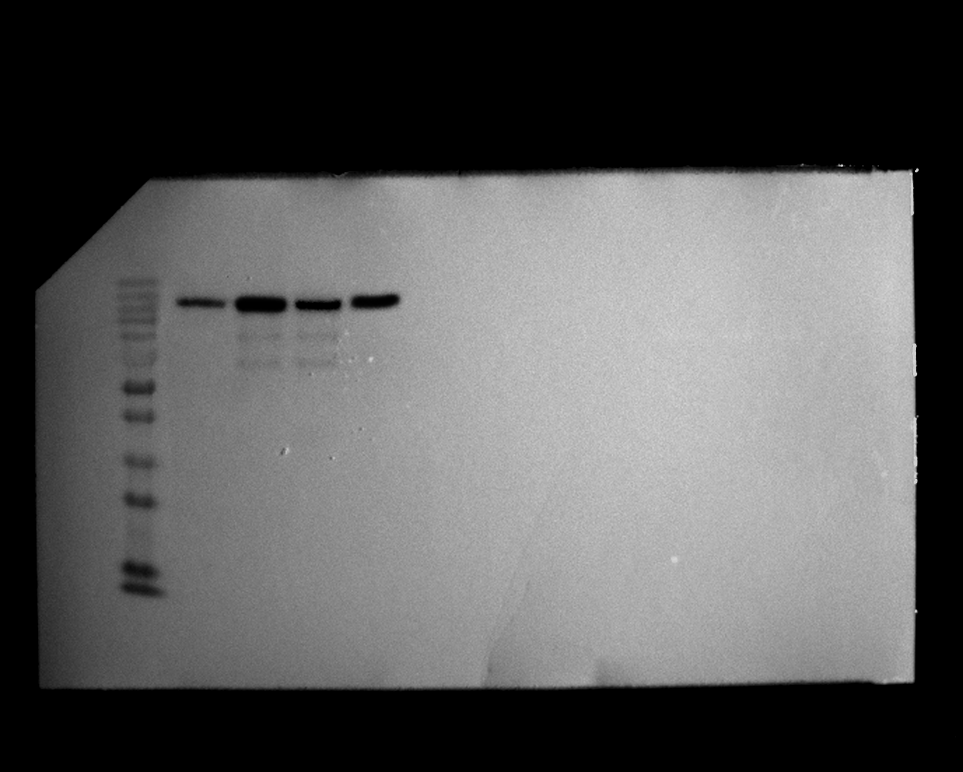

Supplement: S1 Data — (ZIP) [file pone.0338208.s001.zip › YT2021040602-original data/5E/1-ZEB1.tif]

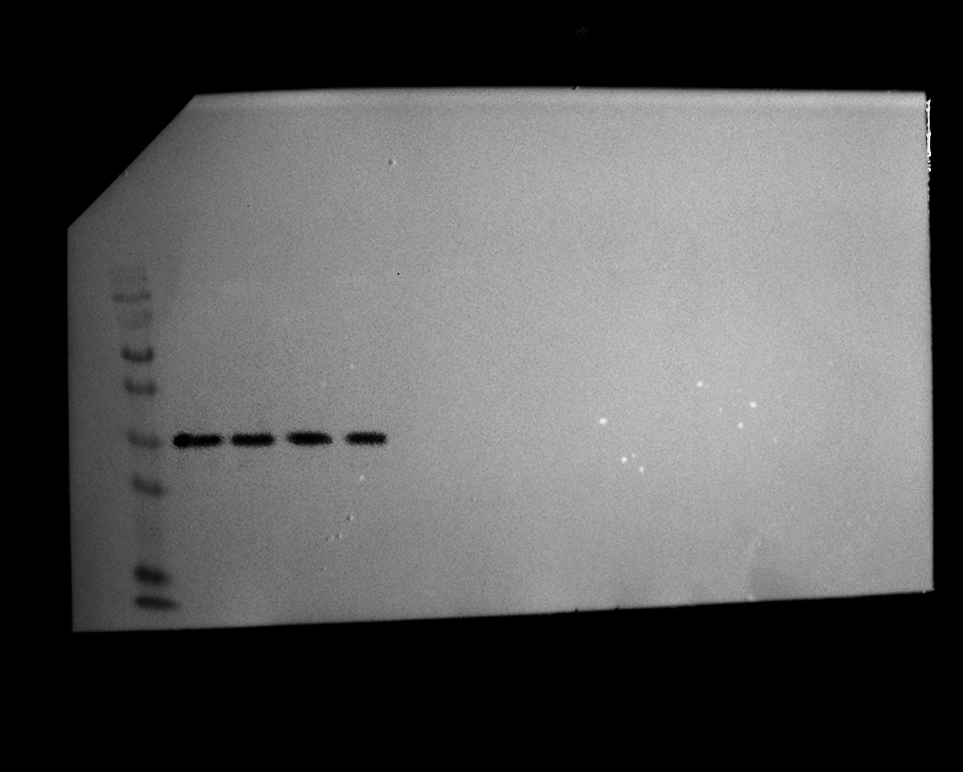

Supplement: S1 Data — (ZIP) [file pone.0338208.s001.zip › YT2021040602-original data/5E/2-GAPDH.tif]

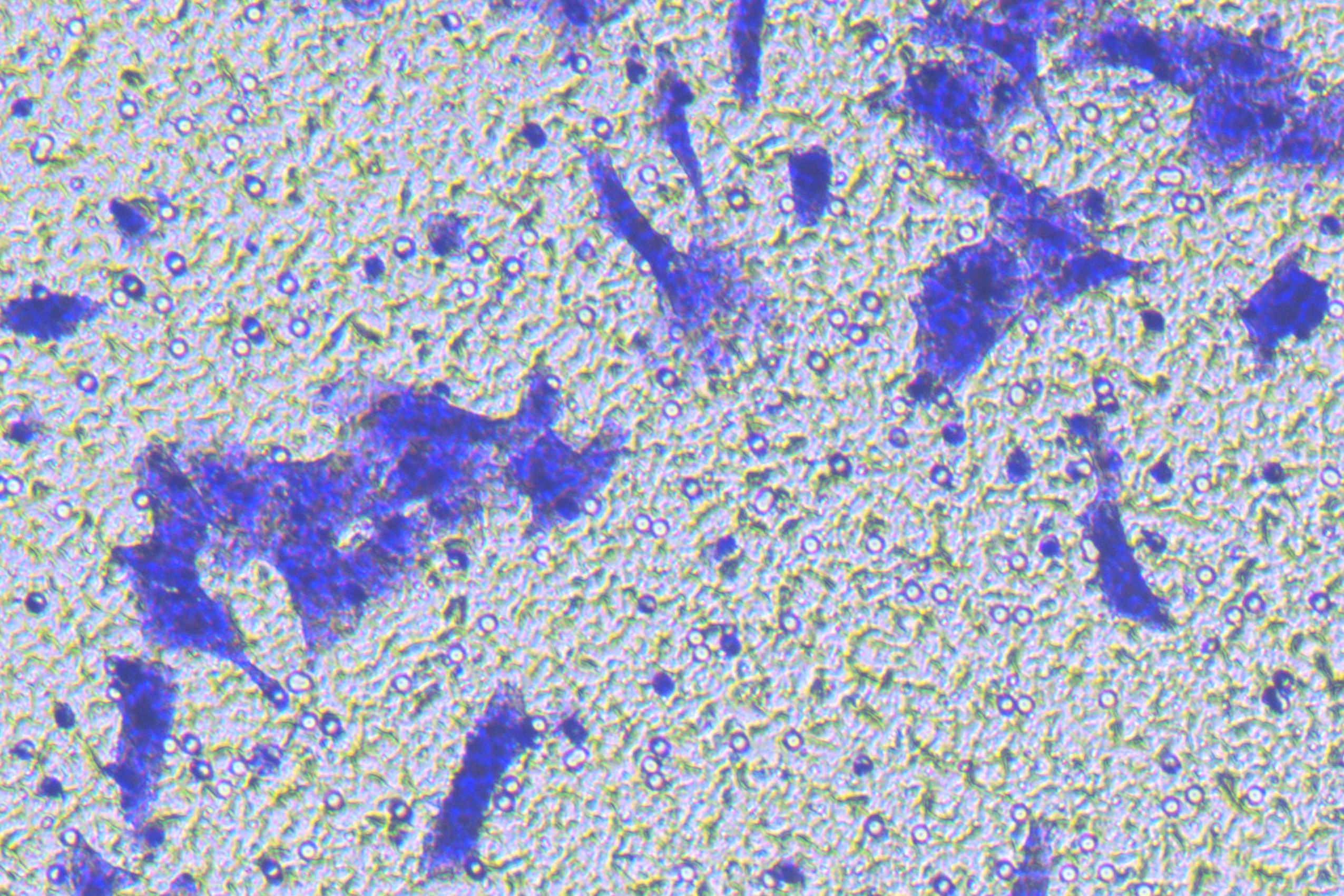

Supplement: S1 Data — (ZIP) [file pone.0338208.s001.zip › YT2021040602-original data/6C/6-1 (1).jpg]

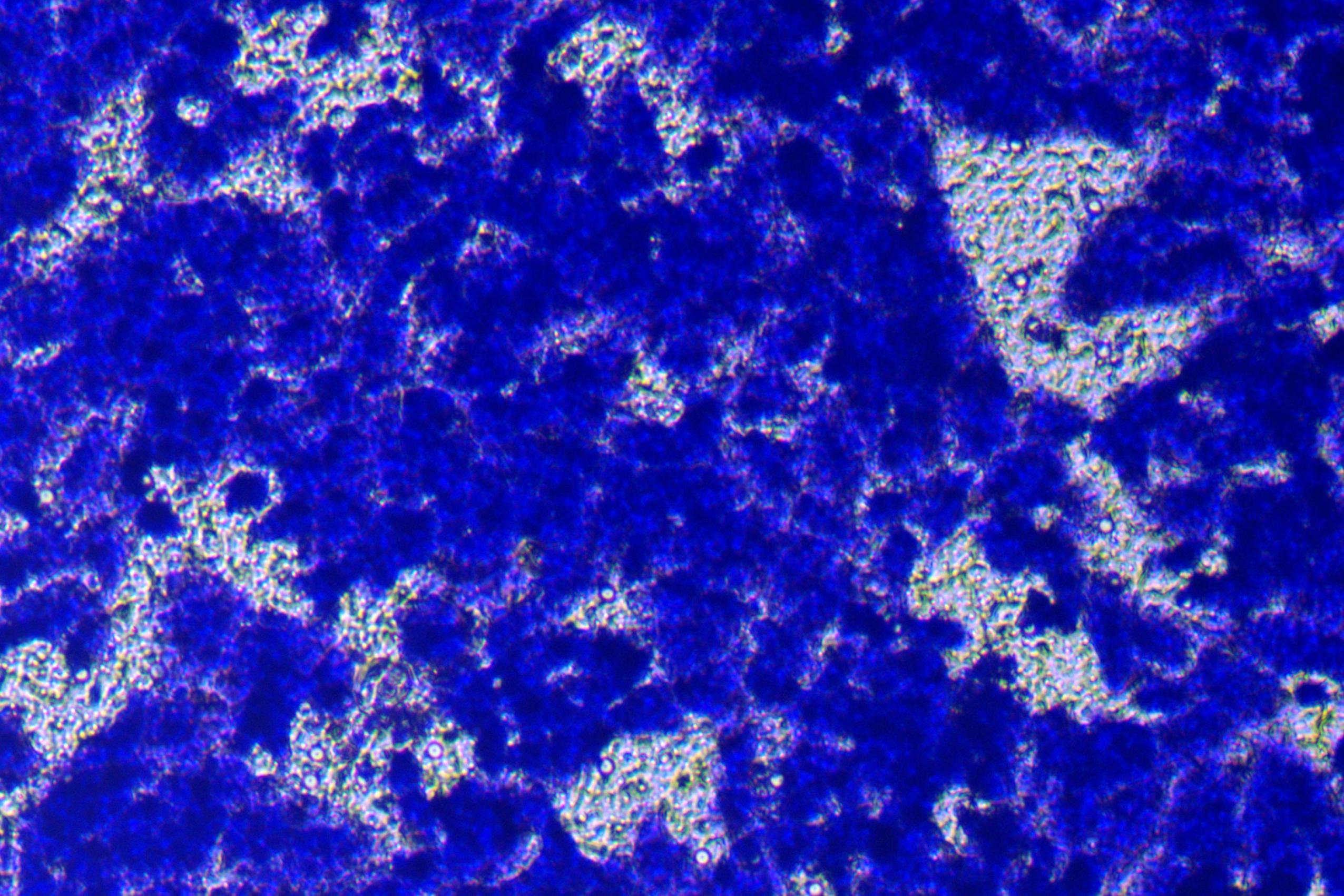

Supplement: S1 Data — (ZIP) [file pone.0338208.s001.zip › YT2021040602-original data/6C/6-1 (2).jpg]

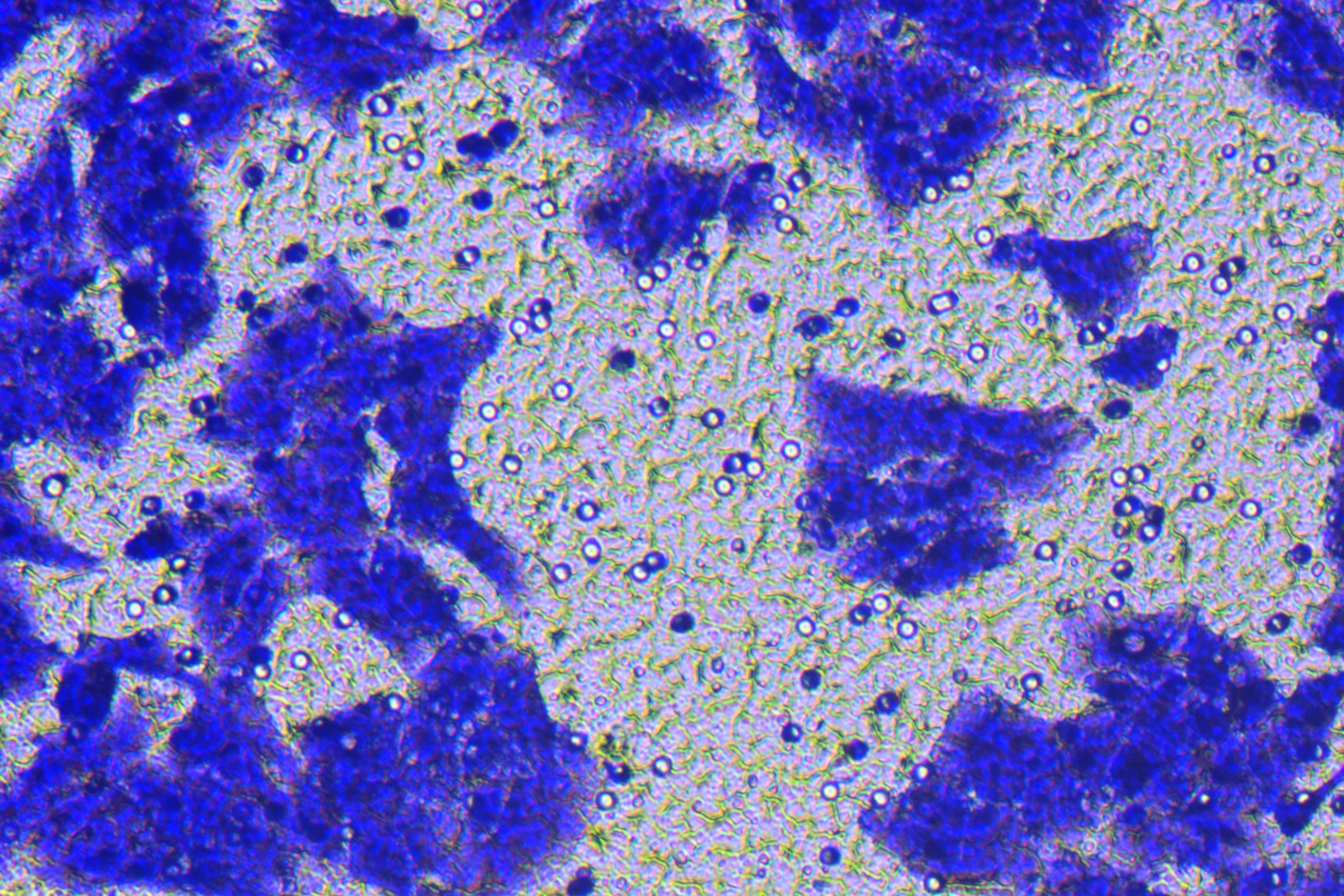

Supplement: S1 Data — (ZIP) [file pone.0338208.s001.zip › YT2021040602-original data/6C/6-1 (3).jpg]

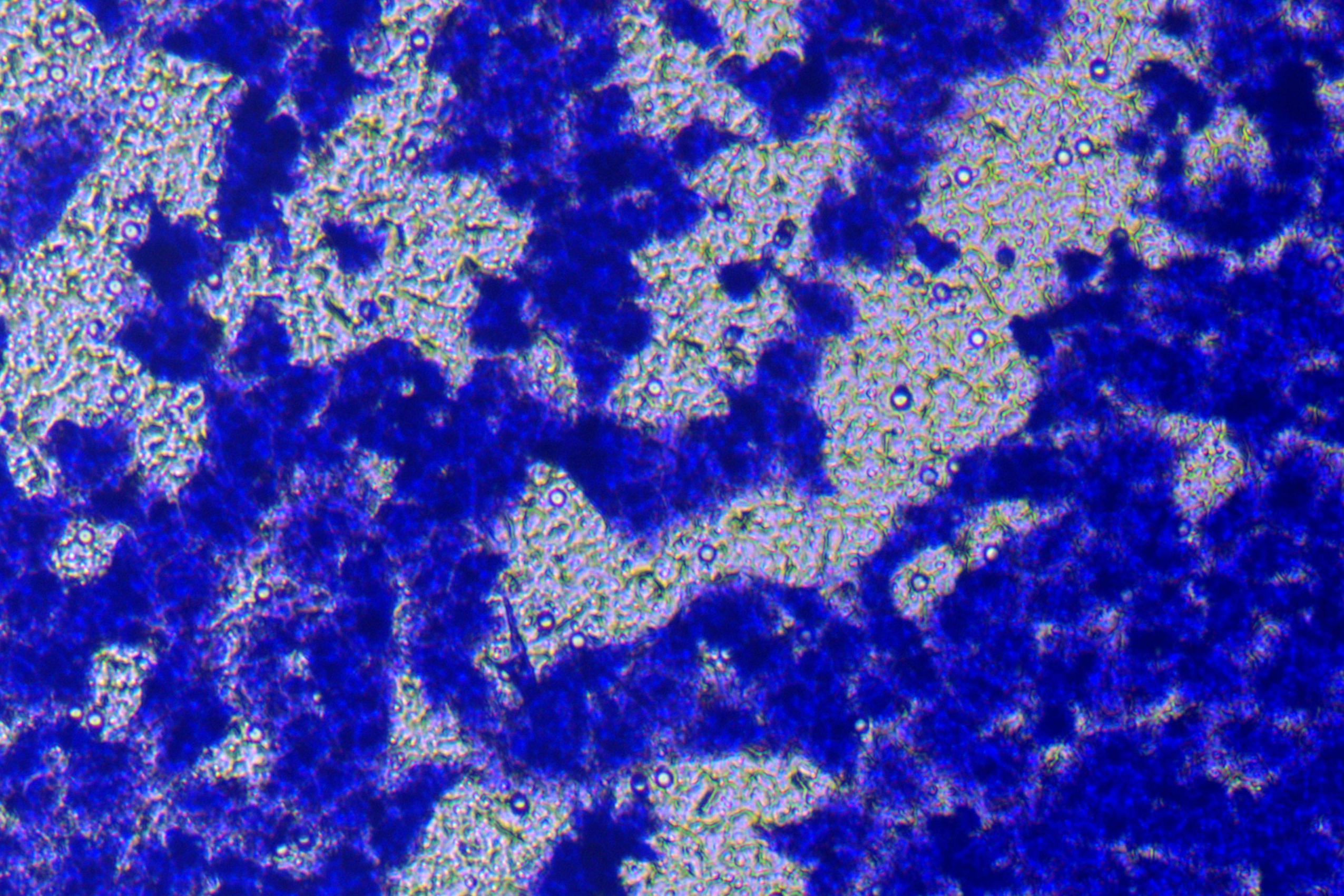

Supplement: S1 Data — (ZIP) [file pone.0338208.s001.zip › YT2021040602-original data/6C/6-1 (4).jpg]

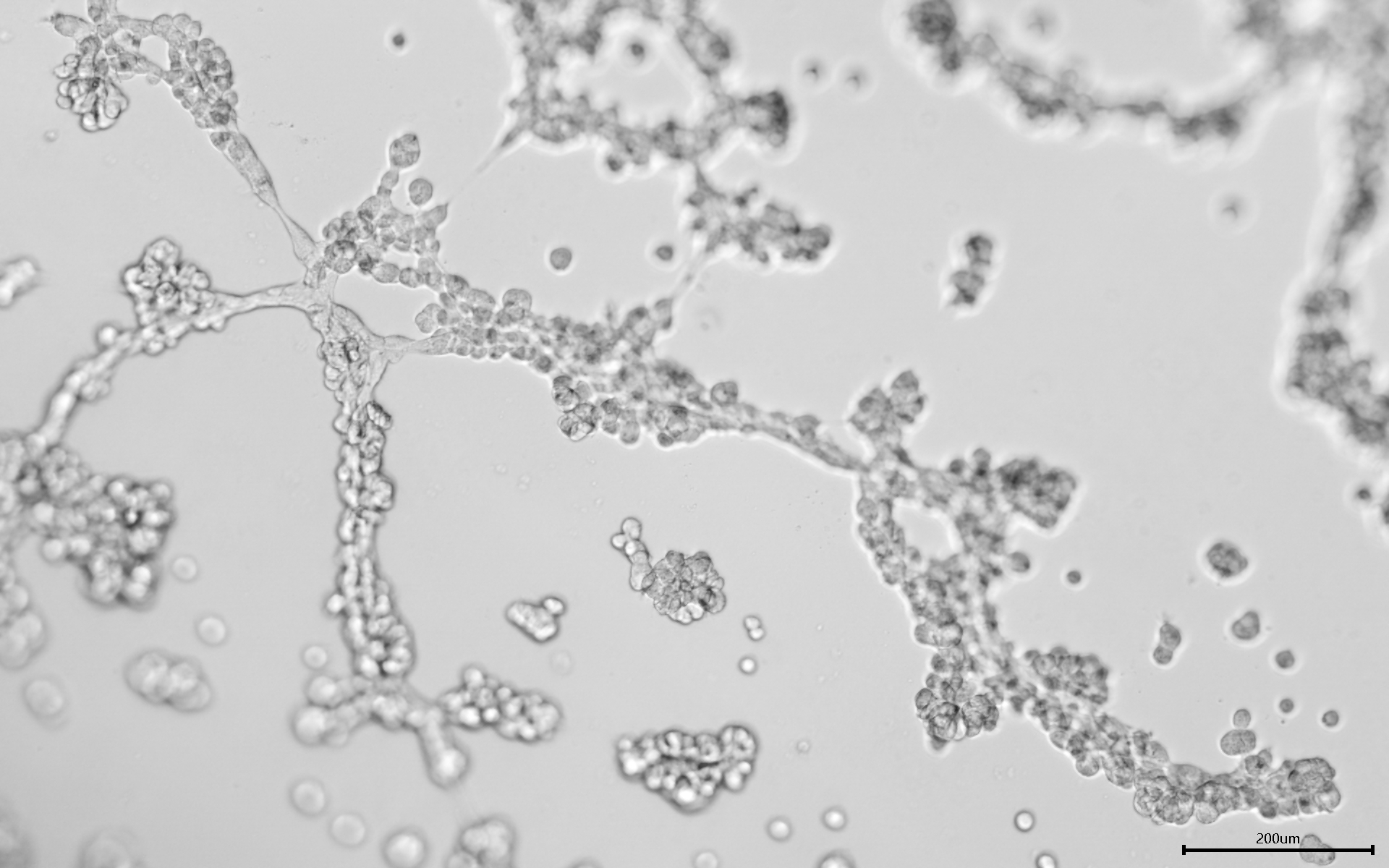

Supplement: S1 Data — (ZIP) [file pone.0338208.s001.zip › YT2021040602-original data/6E/6-1.jpg]

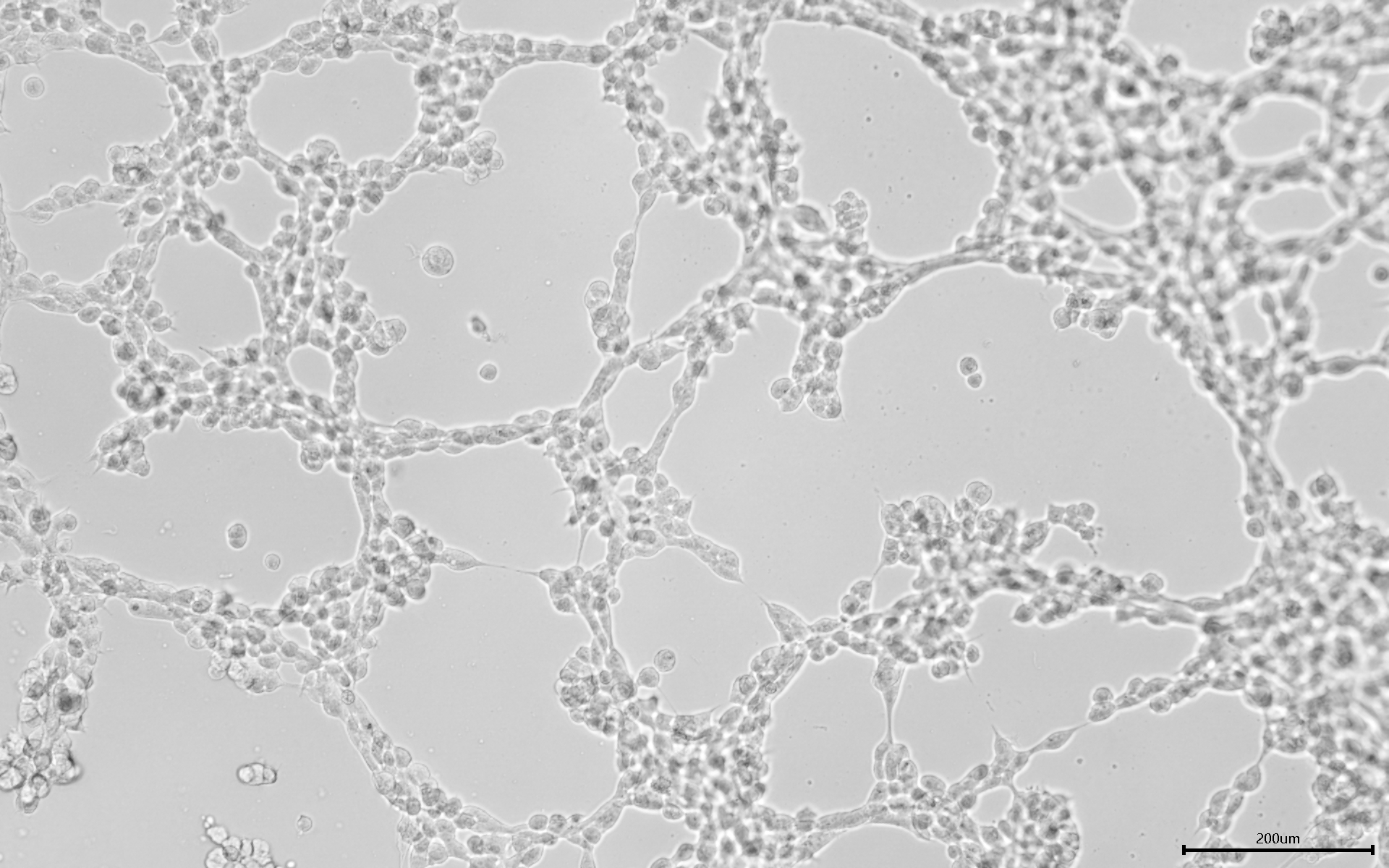

Supplement: S1 Data — (ZIP) [file pone.0338208.s001.zip › YT2021040602-original data/6E/6-2.jpg]

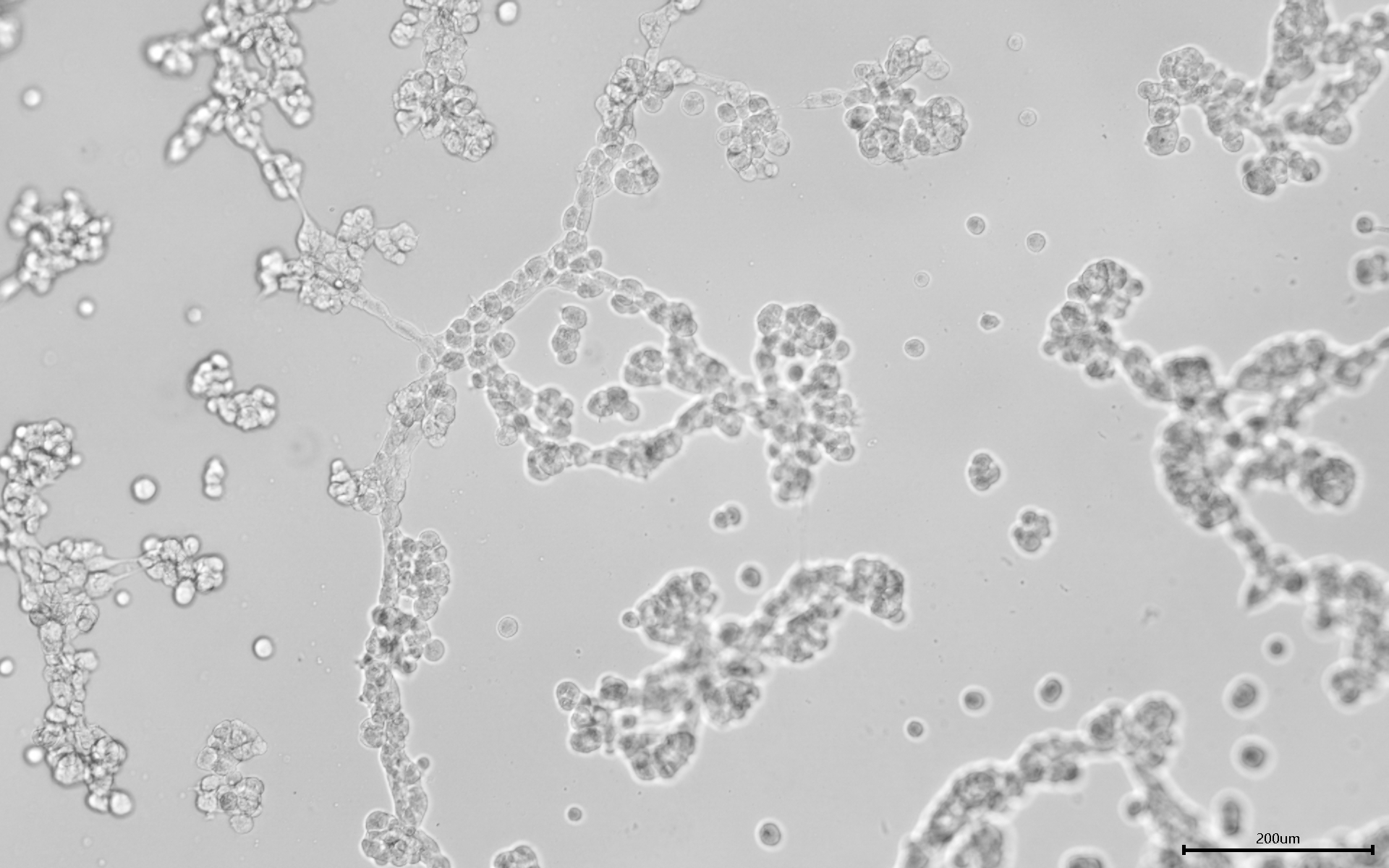

Supplement: S1 Data — (ZIP) [file pone.0338208.s001.zip › YT2021040602-original data/6E/6-3.jpg]

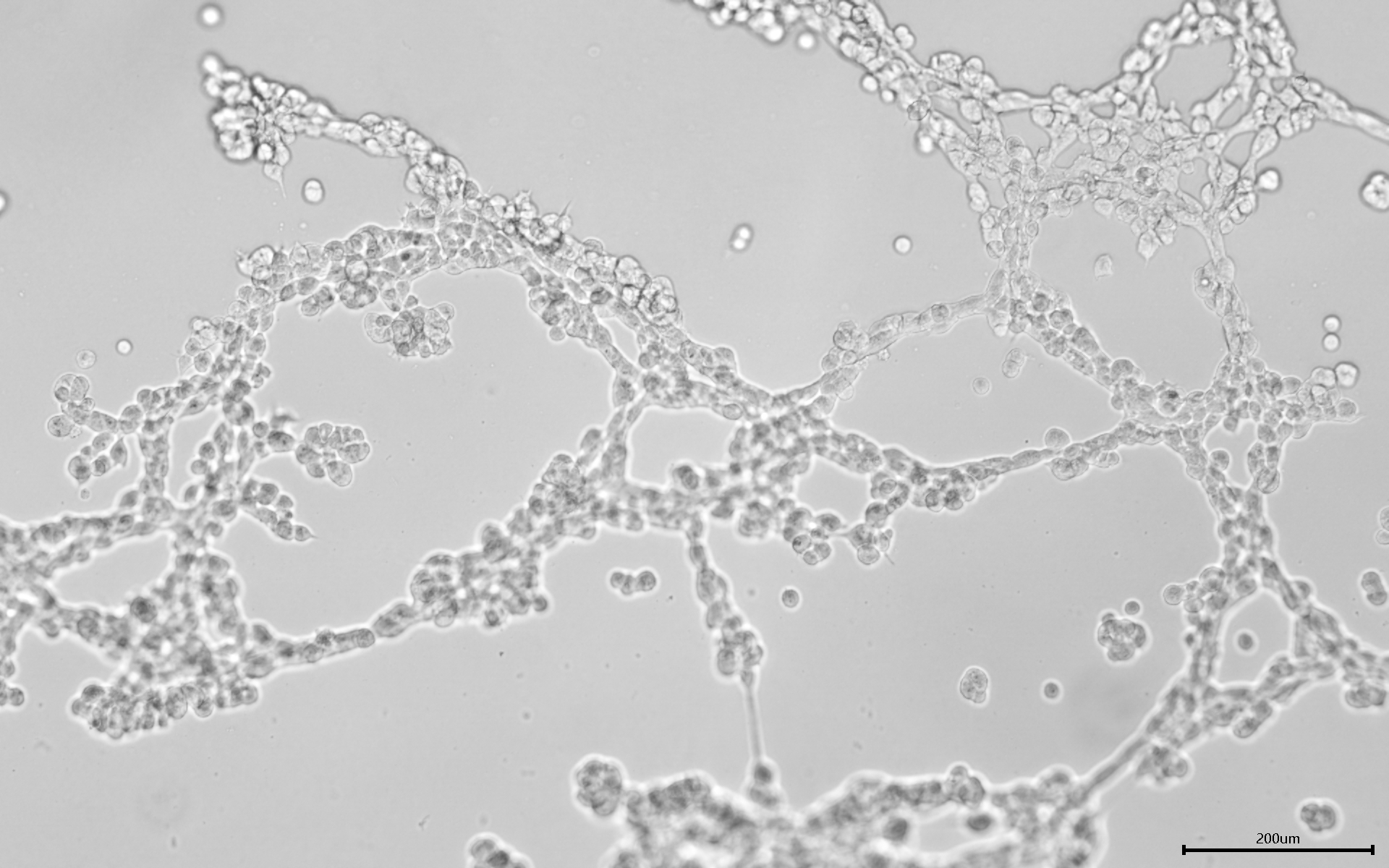

Supplement: S1 Data — (ZIP) [file pone.0338208.s001.zip › YT2021040602-original data/6E/6-4.jpg]
